# Angiogenesis-Related Functions of Wnt Signaling in Colorectal Carcinogenesis

**DOI:** 10.3390/cancers12123601

**Published:** 2020-12-02

**Authors:** Aldona Kasprzak

**Affiliations:** Department of Histology and Embryology, Poznan University of Medical Sciences, Swiecicki Street 6, 60-781 Poznań, Poland; akasprza@ump.edu.pl; Tel.: +48-61-8546441; Fax: +48-61-8546440

**Keywords:** colorectal cancer, Wnt/beta-catenin signaling, angiogenesis, anti-angiogenic therapy

## Abstract

**Simple Summary:**

Angiogenesis belongs to the most clinical characteristics of colorectal cancer (CRC) and is strongly linked to the activation of Wnt/β-catenin signaling. The most prominent factors stimulating constitutive activation of this pathway, and in consequence angiogenesis, are genetic alterations (mainly mutations) concerning *APC* and the β-catenin encoding gene (*CTNNB1*), detected in a large majority of CRC patients. Wnt/β-catenin signaling is involved in the basic types of vascularization (sprouting and nonsprouting angiogenesis), vasculogenic mimicry as well as the formation of mosaic vessels. The number of known Wnt/β-catenin signaling components and other pathways interacting with Wnt signaling, regulating angiogenesis, and enabling CRC progression continuously increases. This review summarizes the current knowledge about the role of the Wnt/Fzd/β-catenin signaling pathway in the process of CRC angiogenesis, aiming to improve the understanding of the mechanisms of metastasis as well as improvements in the management of this cancer.

**Abstract:**

Aberrant activation of the Wnt/Fzd/β-catenin signaling pathway is one of the major molecular mechanisms of colorectal cancer (CRC) development and progression. On the other hand, one of the most common clinical CRC characteristics include high levels of angiogenesis, which is a key event in cancer cell dissemination and distant metastasis. The canonical Wnt/β-catenin downstream signaling regulates the most important pro-angiogenic molecules including vascular endothelial growth factor (VEGF) family members, matrix metalloproteinases (MMPs), and chemokines. Furthermore, mutations of the β-catenin gene associated with nuclear localization of the protein have been mainly detected in microsatellite unstable CRC. Elevated nuclear β-catenin increases the expression of many genes involved in tumor angiogenesis. Factors regulating angiogenesis with the participation of Wnt/β-catenin signaling include different groups of biologically active molecules including Wnt pathway components (e.g., Wnt2, DKK, BCL9 proteins), and non-Wnt pathway factors (e.g., chemoattractant cytokines, enzymatic proteins, and bioactive compounds of plants). Several lines of evidence argue for the use of angiogenesis inhibition in the treatment of CRC. In the context of this paper, components of the Wnt pathway are among the most promising targets for CRC therapy. This review summarizes the current knowledge about the role of the Wnt/Fzd/β-catenin signaling pathway in the process of CRC angiogenesis, aiming to improve the understanding of the mechanisms of metastasis as well as improvements in the management of this cancer.

## 1. Introduction

The Wnt/Frizzled (Fzd)/β-catenin signaling pathway plays a significant role in physiology and pathology (including carcinogenesis) [1,2,3,4]. Since the pioneering mouse model genetic studies and *Drosophila* as well as the discovery of the first mammalian Wnt gene (1982), the role of Wnt signaling was mostly implied in cell growth regulation during embryonic development and maintenance of adult tissue structure [5,6,7]. Aberrant activation of the Wnt/β-catenin signaling pathway as well as its interactions with other pathways is characteristic for various types of carcinogenesis [6,8,9]. 

The elements of canonical Wnt signaling include both a range of extracellular factors (e.g., Wnts) and cytoplasmic proteins (e.g., β-catenin). Wnt ligands, which consist of more than 19 cysteine-rich secreted glycoproteins, mediate cell–cell communication and adhesion, while β-catenin acts as the main downstream effector of the pathway in a target cell [4,9,10,11,12]. The Wnt protein binding cell surface receptor complex is composed of two molecules, the Fzd protein and the single-pass transmembrane molecule, low-density lipoprotein-related protein 5/6 (LPR5/6). There are also several other transmembrane molecules that function as alternative Wnt receptors (e.g., the retinoic acid receptor (RAR)-related orphan receptor (ROR) and related to receptor tyrosine kinase (RYK)) [9]. In turn, there are also Wnt isoforms with the ability to activate the Wnt/β-catenin-independent signaling (e.g., Wnt/calcium and the Wnt/planar cell polarity pathways). Moreover, a number of secreted proteins regulating Wnt signaling have been identified (e.g., Dickkopf (DKK) family proteins, Fzd-related Proteins (FRPs), and Wnt Inhibitory Factor-1 (WIF-1)) [6,9]. 

It was long suggested that the Wnt/Fzd/β-catenin signaling pathway regulates the development of blood vessels in physiological and pathological conditions due to the presence of Wnt ligands (e.g., Wnt-2, -5a, -7a, and -10b), Wnt receptors (e.g., Fzd-1, -2, -3, and -5), and Wnt inhibitors (e.g., FRP-1 and -3) in vascular cells [13]. Descriptions of the biological activity of several identified human Wnt isoforms are already the subject of a number of excellent reviews [1,4,6,9,14]. 

Wnt/β-catenin signaling plays an especially important role in the carcinogenesis of the organs of the gastrointestinal tract in which this pathway takes part in the regulation of embryonic development as well as the homeostasis of adult tissues [1,8,15,16,17]. This group includes colorectal cancer (CRC), the third most commonly diagnosed tumor as well as the second leading cause of cancer-related deaths worldwide [18]. 

The most common clinical CRC characteristics include high levels of angiogenesis, metastasis, and chemoresistance [19]. In CRC etiology, the decisive role is attributed to the genetic changes (especially mutations of tumor suppressor genes and/or proto-oncogenes) occurring in different stages of carcinogenesis (e.g., mutation of the Adenomatous Polyposis Coli (APC) gene during the initiation, and Kirsten Rat Sarcoma Virus (KRAS, K-Ras) gene mutation during the progression of the tumorigenesis) [20]. Currently (2020), a classical *APC-KRAS-TP53* progression model, described by Fearon and Vogelstein in the 1990s [21], has been confirmed, proving that APC mutations have the highest odds of occurring early, followed by *KRAS*, loss of 17p and Tumor Protein 53 (TP53), and SMAD family member 4 (SMAD4) gene mutations [22]. Inactivating mutations of *APC* leads to constitutive activation of Wnt/β-catenin signaling and tumor development. The CRC is therefore considered a prototype example of an oncogenic function of the Wnt/β-catenin signaling [6,8,20]. 

The key component of the Wnt signaling is the cytoplasmic protein β-catenin, serving two important cellular functions. In the cytoplasm, it participates in a so-called destruction complex (DC), together with Axin, APC, and a two serine-threonine kinases: glycogen synthase kinase 3α/β (GSK3α/β) and casein kinase 1 α/δ (CK1 α/δ). Phosphorylation of the β-catenin N terminus represents a pre-requirement for recognition by E3-ubiquitin ligase β-TrCP, with its subsequent degradation in proteasomes. The second important cellular function of β-catenin in epithelial cells is the formation of intercellular junctions of *zonulae adherens* type, together with other catenins (α and γ) and E-cadherin. Activation of the canonical Wnt signaling inhibits β-catenin phosphorylation and protein degradation. Stabilization and cytoplasmic accumulation of β-catenin leads to its transport to the cell nucleus, resulting in the indirect regulation of transcription by the binding of sequence-specific Lymphoid Enhancer Factor/T cell Factor (LEF/TCF) DNA binding factors that upregulate target genes [9,23]. A recent meta-analysis of transcriptomic studies suggests that LEF/TCF-specific transcriptional regulation of Wnt target genes in CRC is relevant for tumor progression and metastasis [24]. It is worth noting that a subset of β-catenin transcriptional targets is LEF/TCF-independent [25]. 

Hence, particular actions of Wnt/β-catenin signaling can be regulated through interactions with various molecular partners including the molecules of adherent junction (E-cadherin), DC elements (axin/conductin, APC, GSK3α/β, CK1 α/δ, and β-TrCP) as well as LEF/TCF family transcription factors [9]. 

The *APC* and catenin β1 (β-catenin) encoding gene (*CTNNB1*) mutations are observed in familial adenomatosis polyposis and 60–90% of sporadic CRC [8,26]. Recently, splice alterations in intronic regions of *APC* and large-frame deletions in *CTNNB1* have been described, increasing Wnt/β-catenin signaling oncogenic alterations to 96% of CRC [27]. Mutations of *APC* encompassing at least two β-catenin downregulating motifs are significantly more frequent in microsatellite unstable (MSI-H) than in microsatellite stable (MSS) CRC [28]. However, the functional effects of *APC* and *CTNNB1* mutations might differ, sparking the search for other factors influencing the action of the Wnt/β-catenin signaling pathway, especially in the context of CRC treatment. 

Several lines of evidence argue for the use of angiogenesis inhibition in the treatment of CRC. In the context of this paper, components of the Wnt pathway with anti-angiogenic activity are among the most promising targets for CRC therapy [6,8,20]. 

This review summarizes the current knowledge about the role of the Wnt/Fzd/β-catenin signaling pathway in the process of CRC angiogenesis for a better understanding of the mechanisms of metastasis as well as improvements in the management of this cancer. 

## 2. Wnt/β-Catenin Signaling and Colorectal Cancer–General Comments

The link between hyperactivation of the Wnt/Fzd/β-catenin signaling and the development of colorectal cancer has been long recognized [2,19,20,29,30]. The activated Wnt/β-catenin signaling promotes CRC cell invasion and migration in vitro, subcutaneous tumor growth, angiogenesis, and liver metastases in vivo [31]. 

The activation of the Wnt canonical pathway causes inhibition of β-catenin phosphorylation as well as the absence of its degradation. Its stabilization and accumulation in the cytoplasm facilitate the transport of β-catenin to the cell nucleus. In the cell nucleus, β-catenin forms a complex with LEF/TCF and intensifies the expression of various target genes associated with proliferation, differentiation, migration, and angiogenesis [2,15,17,30]. In CRC progression and angiogenesis, simultaneous hyperactivation of Wnt/β-catenin signaling and inhibition of the phosphatidylinositol 3’ kinase (PI3K)/Akt pathway promote nuclear accumulation of β-catenin and the Forkhead box 03 protein (FOXO3a), respectively, promoting metastasis by regulating a panel of target genes [2]. Recently, a total of 13 target genes, highly functionally correlated with β-catenin, were identified to be significantly altered in CRC [30]. 

Evaluation of Wnt signaling activity in CRC became a basis to indicate molecular subtypes of this cancer. Hence, based on different responses to epidermal growth factor receptor (EGFR)-targeted therapy (cetuximab), six CRC subtypes were characterized and associated with distinctive anatomical regions of the colon crypts (phenotype), with location-dependent differentiation states and Wnt signaling activity [32]. In another molecular characterization of CRC, four consensus molecular subtypes (CMSs) were indicated including CMS2 (“canonical” subtype) (37%), which is characterized as epithelial and chromosomally unstable with marked Wnt and Myc signaling activation [33]. The most recent classification, among CRC intrinsic subtypes (CRIS), indicates CRIS-D, and to a lesser extent, CRIS-E as subtypes with high Wnt activity and a bottom crypt phenotype [34]. 

## 3. Typical Features of Angiogenesis in Solid Tumors (Including Colorectal Cancer (CRC))

Angiogenesis is one of the key mechanisms of tumor development and is critical for invasive tumor growth and metastasis [31,35,36,37]. The notion that “sustained angiogenesis” is one of the six key processes enabling malignant growth [38], tumor progression, and is one of the commonly accepted indicators of prognosis, is still valid [39]. This process (interchangeably called neoangiogenesis) enables new blood vessel formation through sprouting and splitting from the pre-existing ones. Hence, cancer-focused research currently indicates two major types of angiogenesis: sprouting and nonsprouting (intussusceptive), dependent or independent of endothelial cell (EC) proliferation, respectively [40]. Other authors have reported six mechanisms of vascularization observed in solid tumors. These include, apart from the above-mentioned, recruitment of endothelial progenitor cells (EPCs), vessel co-option, vasculogenic mimicry (VM), and lymphangiogenesis [41]. In CRC, the two main types of angiogenesis (sprouting and nonsprouting) are most commonly described, with the addition of VM [42,43,44]. The “mosaic” vessels have also been reported in the xenograft of human colon adenocarcinoma cells (LS174T) and in human CRC tissues in which both ECs and tumor cells form the lumen. Potential mechanisms of mosaic vessel formation are discussed [45]. 

Among the cells participating in neoangiogenesis/neovascularization in CRC, EPCs, and ECs co-opted from surrounding vessels [41,46,47] as well as cancer stem cells (CSCs) are all indicated [40]. 

The process known as VM is based on the formation of vascular channels without ECs. It is carried out through transdifferentiation of colorectal CSCs (CRCSCs) to form vascular-tube structures (mimic the function of vessels) that facilitate tumor perfusion independently of tumor angiogenesis [40,43,44]. VM formation in CRC is promoted by the Zinc Finger E-box Binding Homeobox 1 (ZEB1) protein. Its silencing resulted in VM inhibition and vascular endothelial (VE)-cadherin downregulation in colon cancer cells (HCT116) [48]. Canonical Wnt signaling also participates in VM. It was demonstrated that in VM-positive CRC samples, the expression of Wnt3a and nuclear expression of β-catenin is increased compared to VM-negative samples. In in vitro (HT29 cells) studies as well as in the mouse xenograft model, the tube-like structure formation was confirmed with the mechanism of overregulated Wnt3a participation in this process explained (through increased expression of vascular endothelial growth factor receptor type 2 (VEGFR-2) and VE-cadherin) [11]. 

The best-known molecular pathway driving tumor vascularization (including CRC) is the hypoxia-adaptation mechanism. When the tumors grow to 0.2–2.0 mm in diameter, they become hypoxic and hindered in growth in the absence of angiogenesis. During the angiogenic switch, pro-angiogenic factors predominate and result in a transition from a vascularized hyperplasia to vascularized tumor, and eventually, to malignant tumor progression [46,49,50,51]. Pro-angiogenic proteins are produced by the tumor and stromal cells and include (i.e., VEGF, transforming growth factor (TGF), basic fibroblast growth factor (bFGF), and platelet-derived growth factor (PDGF)) [35,52,53]. The two latter growth factors are indispensable in the maintenance of the angiogenic process [35]. 

The best-studied pro-angiogenic factor in solid tumors is VEGF, which is important for sprouting angiogenesis as well as the recruitment of circulating EPCs to tumor vasculature [46,47,54]. Several members of the VEGF family have been described, namely the VEGF-A, B, C, D, E and placental growth factor (PlGF, PGF) [50]. These factors bind specific receptors present on the EC surface (VEGFR-1, VEGFR-2, VEGFR-3, neuropilin-1 and -2), which dimerize and activate the intracellular tyrosine kinases (TKs), conducting the angiogenesis promoting signals [41]. VEGF-dependent tumor angiogenesis appears to activate inverse and reciprocal regulation of both VEGFR-1 and VEGFR-2. The VEGFR-1 signaling is required for EC survival, while VEGFR-2 regulates capillary tube formation [55]. 

Increased production of VEGF follows for upregulation of the hypoxia-inducible transcription factor 1 (HIF-1) complex [56,57]. In turn, other factors regulate the HIF-1 complex. An increase in HIF1α expression was reported to be invoked by overexpression of Sine Oculis Homeobox Homolog 4 (SIX4) via Akt signaling. SIX4 also intensified VEGF-A expression by coordinating with HIF-1α in CRC, promoting angiogenesis and tumor growth both in vivo and in vitro [57]. Other pro-angiogenic genes, activated through HIF-1 binding to hypoxia response sequence element (5’-CGTG-3’) in their promoters, are PDGF and TGF-α, activation of which results in blood vessel remodeling and angiogenesis [53,58]. Other HIF-1 target genes with proven roles in colon carcinoma cell invasion include vimentin, keratins 14, -18, -19, fibronectin 1, matrix metalloproteinase 2 (MMP-2), urokinase-type plasminogen activator receptor (uPAR), cathepsin D, and autocrine motility factor (AMF) [58]. In turn, HIF-1α and HIF-2α were proven to play different, or even opposing, roles in canonical Wnt signaling in colon cancer cells. Hence, while HIF-1α silencing negatively affected the stability and transcriptional activity of β-catenin, HIF-2α knockdown did not affect β-catenin level, increasing the transcriptional activity of this protein by inducing its nuclear transport.

Participation of the Wnt/β-catenin axis in CRC angiogenesis is a complex process. It was proven that β-catenin induces VEGF-A expression (mRNA and protein) in human colon cancer cells, underlining the importance of this protein in early and stepwise events of CRC neoangiogenesis [59,60]. Furthermore, VEGF expression positively correlates with cytoplasmic β-catenin expression in tumor cells as well as with tumor progression in vivo [61]. In turn, while VEGFR-1 (Flt-1) is considered specific for ECs, it is also present and functional in different CRC cell lines [62]. Moreover, the study of Ahluwalia et al. reported strong expression of not only VEGF but also VEGFR-1 and VEGFR-2 in human CRC specimens as well as in in vitro studies (HCT116 and HT29 cells). This indicates an autocrine mechanism of action of cancer cell produced VEGF, independent of its primary function in the induction of angiogenesis [63]. Other studies indicate that in CRC, VEGF is secreted through a K-ras/PI3K/Rho/ROCK/c-Myc axis [64]. There are also reports of Wnt signaling promotion by K-ras activation as well as the cooperation of these signaling pathways in the CRC angiogenesis process [59].

In CRC cells, non-endothelial interactions between both VEGF receptor type 1 and 2 (VEGFR-1, VEGFR-2) and the Wnt/β-catenin pathway have also been reported [10,65]. Naik et al. showed that VEGFR-1 is a positive regulator of the Wnt/β-catenin pathway, functioning in a GSK3β-independent manner [65]. Inhibition of VEGFR-1 action by RNA interference (RNAi) or TK inhibitors (TKIs) in Wnt-addicted CRC cells leads to cell death via direct disruption of the Wnt/β-catenin “survival” signaling [10,65].

An interesting model of the regulating influence of Wnt signaling on cancer metabolism and angiogenesis through pyruvate dehydrogenase kinase 1 (PDK1), as a direct Wnt target gene, was demonstrated by Pate et al. They reported that Wnt/β-catenin signaling directs a metabolic program of glycolysis in colon cancer cells (as a common cancer phenotype called the Warburg effect) and affects the tumor microenvironment through increased vessel development [66]. 

When it comes to mechanisms of CRC angiogenesis regulated through Wnt/β-catenin signaling, a growing number of factors promoting or inhibiting this process are described [67,68,69]. 

## 4. Factors Promoting CRC Angiogenesis via Wnt/β-Catenin Signaling

Many described factors promote angiogenesis through Wnt/β-catenin signaling pathway regulation. These include Wnt pathway components and non-Wnt signaling biologically active molecules such as chemoattractant cytokines (chemokines) [70] and various enzymatic proteins including transcription factors [71,72,73,74,75,76,77,78]. The components of the Wnt pathway include agonists (e.g., B cell Lymphoma 9 protein (BCL9)) [67,79,80] as well as antagonists such as the DKK-4 (also called the Dickkopf Wnt signaling pathway inhibitor 4) [81]. An increase in DKK-4 mRNA production was observed in CRC tissues, with elevated ectopic expression of the DKK-4 protein intensifying cell migration and invasion. Moreover, conditioned media from DKK-4 expressing cells also promoted the migrative abilities of CRC as well as the formation of capillary-like tubules of human primary microvascular ECs [81]. 

It needs to be noted that the activity of many classical pro-angiogenic factors (e.g., VEGF-A, MMPs, inducible nitric oxide synthase (iNOS), and chemokines) is usually regulated by at least two signaling pathways (e.g., PI3K/Phosphatase and the Tensin Homolog Deleted on Chromosome Ten (PTEN)/Akt pathway and canonical Wnt/β-catenin downstream signaling). Hence, aberrant Wnt/β-catenin signaling, along with the production of nitric oxide (NO), can positively regulate tumor angiogenesis [68]. 

The BCL9 protein, a transcriptional Wnt/β-catenin cofactor, is the angiogenesis promoting element of the Wnt pathway in CRC [80]. An β-catenin independent function of the BCL9 was also proven, correlating with poor prognosis subtype of the CRC [82]. In the past, it has been underlined that BCL9 intensifies β-catenin-mediated transcriptional activity, independently of Wnt signaling component mutations. BCL9 knockdown enhanced the survival of the xenograft mouse model of CRC and attenuated the expression of pro-angiogenic factors (e.g., CD44, and VEGF), which resulted in a reduction of tumor metastasis and angiogenesis [67]. Hence, BCL9 is a coactivator of the β-catenin-mediated transcription that is highly expressed in tumors, but not in the physiological cells of their origin. The mechanism of BCL9 action in Wnt signaling is based on its direct binding to a unique BCL9-β-catenin binding domain [79], corresponding to its Homology Domain 2 (HD2), which contains a single amphipathic α-helix [83]. 

(C-X-C motif) ligand 8 (CXCL8) (also known as interleukin (IL)-8) is one of the proinflammatory chemokines produced by CRC cells at the tumor invasion front. It promotes angiogenesis through VEGF-A upregulation and cell invasion via the Akt/GSK3β/β-catenin/MMP-7 pathway, by upregulating the anti-apoptotic B-cell lymphoma protein 2 (Bcl-2) [70]. Participation of stromal cell-derived factor 1 (SDF-1) and its receptor (C-X-C chemokine receptor type 4 (CXCR4, fusin, CD184)) was also proven in the mechanisms of CRC progression. In vitro studies confirmed that stromal cell-derived factor 1 (SDF-1) induced CXCR4-positive CRC cell invasion and epithelial-mesenchymal transition (EMT) via activation of the Wnt/β-catenin signaling [84]. 

DEAH box protein 32 (DHX32), one of the RNA helicases, also belongs to the group of angiogenesis promoting enzymes. This transcriptional regulator enhanced the expression of VEGF-A in CRC cells, interacting and stabilizing β-catenin. Thus, the study showed that DHX32 overexpression was associated with angiogenesis in CRC as well as poor outcomes of human CRC patients [72]. Another factor, overexpression of which influences the aggressive phenotype, angiogenesis, chemoresistance, and metastasis of CRC cells, is gankyrin (PSMD10). It is a regulatory subunit of the 26S proteasome complex. A unique pathway participates in the regulation of the above-mentioned processes by gankyrin, namely the PI3K/GSK3β/β-catenin (a cross-talk between the PI3K/Akt and Wnt/β-catenin canonical signaling pathways) [19]. In turn, Cheng et al. proved the stimulating influence on CRC progression and metastasis exhibited by Uba2, a vital component of small ubiquitin-like protein SUMO-activating enzyme, occurring through the regulation of Wnt signaling and EMT enhancement [85]. 

A positive influence on CRC angiogenesis is also attributed to tissue transglutaminase 2 (TGM2). It was reported that silencing of TGM2 inhibited angiogenesis and suppressed the expression of MMP-2, MMP-9, Wnt3a, β-catenin, and cyclin D1 [75]. Similar results were obtained by other authors, describing a decrease in both stemness and angiogenesis through TGM2 inhibition [86]. Similarly, in the case of the Casitas B-lineage lymphoma (c-Cbl) gene encoding CBL protein, which plays a role as an E3 ubiquitin-protein ligase, it was proven that mutant *C-Cbl-Y371H* resulted in augmented Wnt/β-catenin signaling, increasing Wnt gene expression, angiogenesis, and CRC growth. Furthermore, for the regulation of nuclear β-catenin and angiogenesis, phosphorylation of c-Cbl Tyr^371^ is also required [73]. 

High aggressiveness and intense angiogenesis were also attributed to the HCT-116 CRC cells stably overexpressing Akt. In these cells, an increased expression of EMT-related transcription factors was noted including β-catenin. Akt/HCT-116 xenografts were highly aggressive and angiogenic (with high microvessel formation and increased expression of Factor VIII) compared to the pCMV/HCT-116 xenografts. Additionally, the tumors were characterized by the nuclear localization of β-catenin and lower expression of E-cadherin [71]. 

Among the transcription factors, interesting correlations can be observed between Wnt/β-catenin signaling and Zink Finger Transcription Factor Spalt (Sall)-like Protein 4 (SALL4). In a study of the SALL4 gene promoter, a consensus TCF/LEF-binding site within a region of 31 bp was described, possibly playing a role in the stimulation of Wnt/β-catenin signaling in various cancers (including CRC) through direct β-catenin biding and oncogene action [76,78]. In CRC cells, co-expression and correlation between SALL4 and β-catenin expression was described, promoting lymph node metastasis and advanced CRC clinical stage [78]. Recently, it was also demonstrated that SALL4 participates in the process of human umbilical vein ECs (HUVECs) angiogenesis, modulating VEGF-A expression [87]. 

When it comes to other transcription factors, it was demonstrated that the Forkhead Box Q1 protein (FOXQ1) protein is overexpressed in CRC and correlates with stage of tumor and lymph node metastasis. Small iRNA knockdown experiments on the SW480 cell line weakened the aggressive potential of cancer, downregulating angiogenesis, invasion, EMT, and resistance to drug-induced apoptosis through the inhibition of nuclear translocation of β-catenin. It was also demonstrated that the expression and action of FOXQ1 were promoted by TGF-β1. Hence, CRC progression via angiogenesis was enabled by the co-operation of two signaling pathways: Wnt and TGF-β1 [77]. 

The influence of several plant-based compounds on CRC angiogenesis [88] as well as the connection between the activity of such compounds and Wnt/β-catenin signaling in CRC, were also investigated [89]. These compounds include the water solutions of *Aloe vera* extracts (with two active components: aloin and aloesin). It seems that the action of active *Aloe Vera* components on angiogenesis and tumor growth depends on the activity of more than one signaling pathway. It was proven that aloin promotes activation of the Wnt/β-catenin signaling as well as inhibits the Notch signaling pathway in CRC cells only in the presence of Wnt3a. In turn, aloesin directly activates Wnt signaling and inhibits the Notch pathway in a Wnt3a independent manner [89]. These results are contradictory to previous reports describing the inhibiting influence of aloin on CRC angiogenesis via signal transducer and activator of transcription protein 3 (STAT3) activation [88]. 

## 5. Factors Inhibiting CRC Angiogenesis via Wnt/β-Catenin Signaling

Furthermore, various factors inhibiting angiogenesis CRC via Wnt/β-catenin signaling were also described. These include antagonists of Wnt (e.g., DKK-1 genes) [90]. DKK-1 protein expression in CRC tissues was downregulated during the CRC adenoma-carcinoma sequence, correlating with the downregulation of VEGF expression and decreased microvessel density. Overexpression of DKK-1 in CRC cells in vitro (HCT116) inhibited the formation of tube-like structures and downregulated VEGF expression in HUVECs. Xenografts of DKK-1 overexpressing CRC cells have decreased microvessel density (MVD) and VEGF expression vs. the control cells [90]. 

Angiogenesis inhibiting factors also include tumor suppressors (e.g., tumor necrosis factor α (TNFα)-induced protein 8 like 2 (TIPE2, TNFAIP8L2)) [91]. TIPE2 plays a role in immune homeostasis and is associated with carcinogenesis on many tumors [92]. The study by Wu et al. on human rectal adenocarcinoma demonstrated that the expression of this protein was higher in tumor tissues compared to the control. However, TIPE2 overexpression increased cell apoptosis through downregulation of Wnt3a, phospho-β-catenin, and GSK3β expression in rectal adenocarcinoma cells. It was proven that TIPE2 knockdown promoted the growth of this tumor through angiogenesis modulation. The participation of TIPE2 in the regulation of proliferation, migration, invasion, and, consequently, angiogenesis involves the Wnt/β-catenin and TGF-β/Smad2/3 signaling pathways [91]. 

A relation between re-expression of type 1 cyclic guanosine monophosphate (cGMP)-dependent protein kinase (PKG) in metastatic colon carcinoma and reduced tumor angiogenesis was also described. In vivo studies confirmed reduced levels of VEGF in PKG-expressed tumors compared with tumors that were derived from parental SW620 cells. Moreover, PKG expression was associated with reduced levels of β-catenin in comparison with the parental cells. Administration of exogenous PKG in SW620 cells also inhibited the expression of β-catenin and resulted in a decrease of TCF-dependent transcription [93]. 

Another molecule inhibiting β-catenin mRNA production and promoter activity is the scaffold/matrix attachment region binding protein 1 (SMAR1). Effects inhibiting the Wnt/β-catenin signaling activity were obtained by recruiting histone deacetylase-5 to the β-catenin promoter, resulting in decreased CRC cell migration and invasion as well as indirectly inhibiting cancer progression and angiogenesis. Moreover, smaller tumor size in in vivo NOD-SCID mice correlated with the suppression of β-catenin [94]. 

Protein kinase C-α (PKCα) can also function as a Wnt/β-catenin inhibitor, participating in RORα phosphorylation, hence inhibiting transcriptional activity of β-catenin. The key mechanism of that Wnt/β-catenin signaling inhibition is Wnt5a/PKCα-dependent phosphorylation on SER^35^ of RORα. Reduction of RORα phosphorylation in >70% of CRC cases appears clinically important, together with a significant correlation of this reduction and PKCα phosphorylation in tumor samples compared to normal tissue specimens [95]. It was also proven that PKCα also phosphorylates β-catenin itself, leading to its physiological degradation in proteasomes [96]. Recent in vitro (DLD-1 cells incubated with PKCα activators) and in vivo (C57BL/6J mice) studies with knocked-out *PRKCA* (gene encoding mouse PKCα) confirmed that this kinase exerts an anti-tumor (anti-growth, stimulating cell death) effect on cancer cells [97]. 

Similarly, an inhibitory influence of certain plant-based compounds (known and used in traditional Chinese medicine) on angiogenesis is described. The research indicates the inhibiting function of sporamin (a Kunitz-type trypsin inhibitor, found in sweet potato (*Ipomea batatas*)) on the number and mass of tumor nodules formed in the abdominal cavity via reduction of β-catenin (mRNA and protein) and VEGF concentration in the liver of mouse xenografted with LoVo CRC cells [97]. Another such compound is Tanshinone IIA (Tan IIA, TSA), the active lipophilic component of a Chinese *Salvia miltiorrhiza Bunge* plant. The mechanism of its action in normoxic and hypoxic microenvironment conditions is based on the inhibition of TGF-β secretion via inhibition of HIF-1α, which drives angiogenesis by promoting β-catenin nuclear translocation and TCF/LEF activation [98]. 

A significant influence on Wnt/β-catenin signaling and downregulation of the key genes: TCF4 (transcription factor 7-like 2, TCF7L2), cyclin D1, and c-Myc in CRC are also exerted by emodin (the anthraquinone-active substance) [99,100]. This active component of the roots and bark of several plants regulates the expression of key components of Wnt signaling, namely β-catenin and TCF7L2 as well as several downstream targets of this pathway. Additionally, two new targets of emodin action, the p300 Wnt co-activator (downregulated), and the HMG-box transcription factor 1 (HBP1) repressor (upregulated) were indicated in CRC cell lines [99]. Recent research confirmed these observations through the demonstration of EMT and tumor growth inhibition. After emodin administration, a decrease in the expression of MMPs (MMP-7 and MMP-9), VEGF, N-cadherin, Snail, and β-catenin was observed together with an increase in E-cadherin mRNA expression [100]. 

A recent study (2020) indicated the inhibitory influence of 6-Gingerol (6-G) on mouse CRC tumorigenesis and angiogenesis, with the participation of the Wnt/β-catenin signaling [101]. The use of ginger (*Zingiber officinale*) extract and 6-G in therapy against cancers (including CRC) is very well known in medicine (reviewed in [102]). After 6-G exposition, downregulation of various oncogenic proteins’ expression was demonstrated, including Wnt3a and β-catenin. Inhibition of angiogenesis occurred through the downregulation of the concentration of VEGF, Angiopoietin-1 (ANG-1), FGF, and growth differentiation factor 15 (GDF-15) in the colon of benzo[a]pyrene and dextran sulfate sodium (DSS)-exposed mouse [101]. 

Furthermore, the inhibitory action of Raddeanin A (RA), an active oleanane type triterpenoid saponin and a major compound isolated from *Anemone raddeana Regel* was also described in CRC, influencing invasion and metastasis of this cancer’s cells. This process occurred via nuclear-factor kappa B (NF-κB) and STAT3 signaling pathways. However, the main signaling pathway associated with RA action seems to be the PI3K/Akt (reviewed in [103]). Inhibition of cell proliferation and tumor growth occurs through the downregulation of canonical Wnt/β-catenin and NF-κB signaling pathways. In the mechanism of Wnt pathway downregulation, suppression of phosphorylated lipoprotein-related protein 6 (p-LPR6), Akt inactivation, the release of GSK3β inhibition, and attenuation of β-catenin expression were noted [104]. It was proven that RA inhibits HUVEC proliferation, motility, migration, and tube formation as well as reduces angiogenesis in the chick embryo chorioallantoic membrane. As an anti-tumor plant-based compound, RA also inhibits angiogenesis in vitro (HCT-15 cell line) as well as in preclinical models in vivo. Mechanism of its action in CRC is based on the modulation of VEGF-mediated phosphorylation of VEGFR-2 as well as downstream focal adhesion kinase (FAK), phospholipase C γ1 (PLCγ1), Src, and Akt kinases [105]. 

Pan et al. demonstrated the inhibitory action of aloin (derived from *Aloe barbadensis Miller* (*Aloe vera*) leaves) on angiogenesis, mainly occurring through the inhibition of the STAT3 signaling pathway. Aloin inhibited HUVEC proliferation, migration, and tube formation in vitro as well as activation of VEGFR-2 and STAT3 phosphorylation in ECs. After aloin administration in SW620 CRC cells, a downregulation of antiapoptotic (Bcl-xL), pro-proliferative (C-Myc), and angiogenic factors (e.g., VEGF) was also observed. Moreover, reduced tumor volumes and weight were noted in vivo (mice xenograft model) [88]. 

The activity of other plant-derived compounds as potential therapeutic targets will be discussed in further sections of this review. 

In Table 1, a summary of pro- and anti-angiogenic activity of chosen factors influencing the Wnt/β-catenin signaling in CRC is presented. 

## 6. Cellular Components of Tumors in Angiogenesis-Related Functions of Wnt/β-Catenin Signaling in CRC

**Figure 1:**
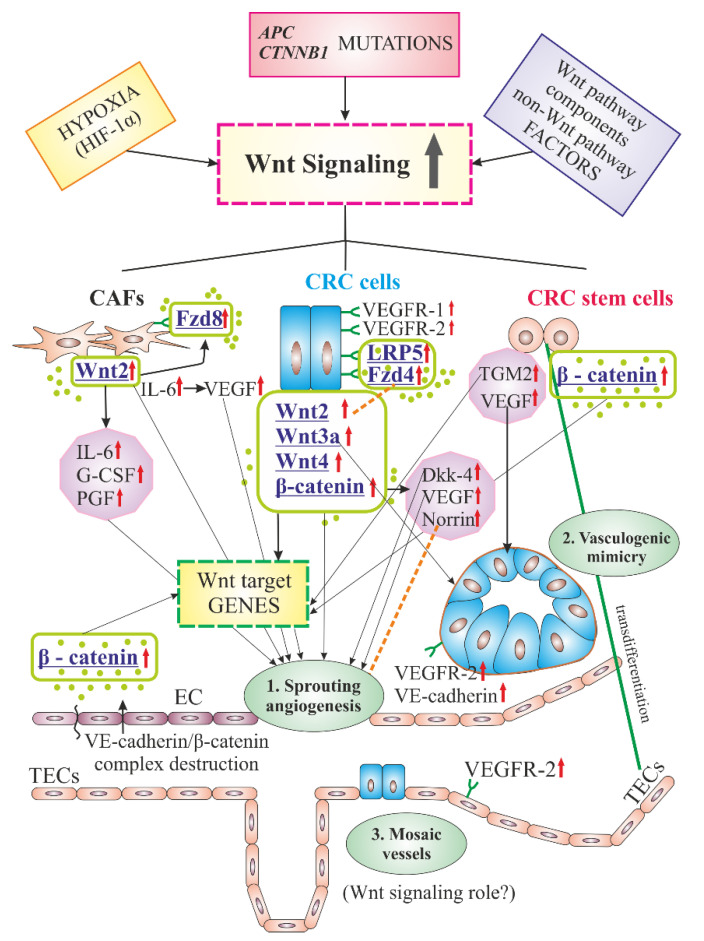
Angiogenesis-related functions of the Wnt/β-catenin signaling pathway in colorectal cancer (CRC). Schematic overview of the main components of the canonical and non-canonical Wnt signaling overexpressed (↑) in the main cells of the tumor (i.e., CRC cells, CRC stem cells, and cancer-associated fibroblasts (CAFs)). Various extracellular factors (e.g., Wnts) and cytoplasmic proteins (e.g., β-catenin) secreted by these cells play a stimulating (arrows) or regulating (dotted lines) role in angiogenesis. There are several other pro-angiogenic factors (e.g., VEGF, IL-6, Norrin) that interact with the Wnt pathway components to enhance angiogenesis in CRC. CRC stem cells can directly transdifferentiate into tumor endothelial cell (TECs) to form vascular-tube structures (vasculogenic mimicry). In the sprouting angiogenesis and vasculogenic mimicry, Wnt pathway-related mechanisms are well described. In turn, the role of Wnt signaling in mosaic vessel formation in CRC is poorly understood (for details see text). Abbreviations: APC—Adenomatous Polyposis Coli gene; CTNNB1—catenin β1 (β-catenin) gene; DKK—Dickkopf-related Protein; ECs—Endothelial Cells; Fzd4,8—Frizzleds 4,8 proteins; G-CSF—Granulocyte Colony-stimulating Factor; HIF-1α—Hypoxia-inducible Factor 1 α; IL-6—Interleukin 6; LRP5—Low-density Lipoprotein-related Protein 5; PGF—Placental Growth Factor; TGM2—Tissue Transglutaminase 2; VE-cadherin—Vascular Endothelial cadherin; VEGF (R)—Vascular Endothelial Growth Factor (Receptor).

Cells active in CRC angiogenesis mediated by Wnt/β-catenin signaling (interacting with vascular ECs) are tumor colorectal cells [109], CRC stem cells [40,64,110,111] and CRC-associated fibroblasts [35,64,106] (Figure 1). The group of cells crucial in the process of angiogenesis and metastasis promotion includes those directly associated with blood vessels, namely progenitor ECs (EPCs) [35,112], tumor-associated ECs (TECs) [37], pericytes [113], and platelets [35,53]. 

### 6.1. Tumor Cells

β-catenin produced by the tumor directly induces VEGF production and an increase in vessel density, which was proved in the *Min/+* mouse model. Levels of VEGF-A (mRNA and protein) upregulated by 250–300% were observed in an in vitro model, using transfection of normal colon epithelial cell line NCM460 with activated β-catenin. The relation between β-catenin and regulation of VEGF-A expression was also proven on colon cancer cell lines (HCT116, SW620), which indicates the participation of β-catenin in angiogenesis initiation. A positive correlation was demonstrated between the upregulation of VEGF-A expression and *APC* mutational status [60]. 

Wnts are not the only ligands of the Fzd receptors. Norrin, a non-Wnt ligand, binds selectively to Fzd4 and stimulates Wnt signaling [9]. The norrin/Fzd4 interactions are modulated via the regulation of Fzd4 expression by Wnt2 [114]. Norrin produced by colon cancer cells increases EC growth and motility in a tumor microenvironment [114,115]. In turn, ECs in the microenvironment of colorectal tumor comprise all of the components of the Norrin signaling pathway. Hence, this signaling pathway has an important role in CRC tumor microenvironment angiogenesis [115]. 

In CRC cells, aberrant expression of E-cadherin/β-catenin complex can be observed as well as that of other angiogenesis markers such as Syndecan-1, platelet (Endothelial) cell adhesion molecule 1 [P(E)CAM-1, CD31], and endoglin (CD105), all involved in tumor progression and prognosis. Moreover, endoglin expression in tumor cells was positively correlated with E-cadherin, β-catenin, and Syndecan-1 [116]. 

It was demonstrated that exosomes derived from hypoxic CRC cells promote angiogenesis. These exosomes, enriched with Wnt4, promoted the proliferation and migration of ECs through Wnt4-induced β-catenin signaling. It was proved that Wnt4 increased nuclear translocation of β-catenin in ECs. Furthermore, an increase in tumor size and angiogenesis via CRC cell-derived exosomes was also confirmed in an animal in vivo model [109]. 

### 6.2. Colorectal Cancer Stem(-Like) Cells (CRCSCs)

CSCs of human CRC are unique cell types able to maintain tumor mass, modify the tumoral microenvironment by expressing angiogenic factors and enhanced neovascularization, and survive outside of the primary tumor at metastatic sites [40,64,110,111]. These cells play an important role in tumor vasculogenesis through their ability for transdifferentiation into human colorectal carcinoma ECs as well as to generate functional blood vessels [110]. Moreover, they also play a role in VM [40]. Surface markers of CSCs have been characterized, and their role in angiogenesis of all gastrointestinal cancers (including CRC) has been discussed in great detail in recent reviews [40,111]. Furthermore, it seems that CRCSCs cooperate with pericytes during angiogenesis initiation in CRC [113]. 

The mutual relations between CRCSCs and the canonical Wnt/β-catenin signaling pathway are also described in the case of CRC. This signaling pathway is a master regulator of a balance between stemness and differentiation in several adult stem cell niches including colon CSCs population in intestinal crypts of Lieberkühn. The colon-crypt base is characterized by high activity of Wnt signaling, especially in the bottom third of the crypts (where CSCs reside) due to signals from the stromal microenvironment cells [1,32]. In HCT116 and HT29 sphere models, Wei et al. demonstrated the promotion of proliferation, migration, and tube formation of EPCs via VEGF secretion by spheroid cells [112]. The malignancy in CRC spheroid cells (with high CSC characteristics) was associated with increased expression of TGM2 (TG2), β-catenin, VEGF, and EMT features [86]. Many new canonical Wnt signaling gene targets on CRCSCs were also identified as components of the stem-like subtype signature described by the authors [32]. 

### 6.3. Cancer-Associated Fibroblasts (CAFs)

Cancer-associated fibroblasts, as a major component of tumor stroma, play an underestimated role in the development and progression of various solid tumors (including CRC) [117,118]. Activated CAFs isolated from CRC produce IL-6, which induces angiogenesis mainly through intensification of VEGF-A expression in these cells [119]. Among the pro-angiogenic Wnt signaling components highly enriched in colorectal cancer CAFs is the Wnt2 protein [106,107,108,120]. Initially, overexpression of this protein was demonstrated in CRC cells, with a knockdown of Wnt2 downregulating Wnt/β-catenin target gene expression. Furthermore, the pro-proliferative properties of this protein were also observed [121]. The role of CAFs, as the main source of Wnt2 in CRC, was first demonstrated by Kramer et al. [107]. CAF-derived Wnt2 activates canonical signaling in APC/β-catenin wild-type colon cancer cells (but not in cells with *APC*/*CTNNB1* mutations) in a paracrine manner. Fzd8, a putative Wnt2 receptor, was identified on CAFs. It was demonstrated that Wnt2 activates autocrine canonical Wnt signaling in primary fibroblasts, which was connected to the pro-migrative and pro-invasive phenotype. These studies indicate the major role of Wnt2 in the promotion of growth, invasion, and CRC metastasis in vivo [107]. Further research of this group demonstrated that Wnt2 intensifies EC migration and invasion. However, induction of the canonical Wnt pathway was only observed in a small number of cells. In turn, in the CRC xenograft model, Wnt2 overexpression led to enhanced vessel density and tumor volume. A correlation of Wnt2 levels was observed with the expression of vascular markers as well as an increase in pro-angiogenic functions of many proteins (e.g., ANG-2, IL-6, granulocyte colony-stimulating factor (G-CSF), and placental growth factor (PGF)). Three of them (IL-6, G-CSF, and PGF) have a major part in angiogenesis intensification via increased Wnt2. Hence, the authors proved the key role of Wnt2 in the formation of the active CAF phenotype in CRC, associated with the maintenance of pro-angiogenic secretome and extracellular matrix (ECM) remodeling signals [106]. The research of Aizawa et al. demonstrated that gene sets related to the Wnt signaling were highly expressed in CAFs (with Wnt2 specifically expressed). The authors observed Wnt2-induced cancer cell migration and invasion in CRC and confirmed the correlation between Wnt2 expression and clinicopathological data (including venous invasion) in CRC in vivo studies [108]. 

### 6.4. Tumor-Associated (Vascular) Endothelial Cells (TECs, TVECs)

In physiology, ECs are responsible for the formation of a semi-permeable barrier, a process enabled by the structure of intercellular connections as well as the presence of VE-cadherin (cadherin 5/CD144) and β-catenin, linking the VE-cadherin junction complex to the cytoskeleton [122,123]. The factors destroying intercellular connections in ECs also play a role in angiogenesis induction. Temporary and reversible damage of the VE-cadherin/β-catenin junctional complex was observed as a result of the activity of some inflammatory agents (e.g., histamine) [122]. A decrease in VE-cadherin expression, release of β-catenin from the complex, induction of nuclear accumulation of β-catenin, and an increase in MMP-7 mRNA expression in HUVECs were also observed after application of recombinant matrilysin (MMP-7) [124]. 

In tumor (including CRC) blood vessels, structural and functional changes can be observed, connected to alterations in leukocyte trafficking. It was demonstrated that VE-cadherin expression and downstream activation of the Akt/GSK3β/β-catenin signaling caused an increase in the expression of the chemokine (C-C motif) ligand 2 (CCL2) and CXCL10, which facilitate CD8+ T cell transmigration into tumor parenchyma. Restoration of proper EC junctions not only inhibits vascular leak, but also regulates immune cell infiltration into tumors [125]. The endothelial Wnt/β-catenin signaling also participates in angiogenesis through differentiation and sprouting of ECs, remodeling as well as arterio-venous specification [126,127]. 

The blood vessels produced within the tumor are lined by TECs, characterized by abnormal proliferation and apoptosis [35]. TECs exhibit many altered phenotypes compared with normal ECs and produce several “angiocrine factors”, which promote tumor progression. One of these factors is biglycan, which is produced in highly metastatic tumors including CRC. Stages and mechanisms of tumor metastasis involving TECc as well as elements of the stromal microenvironment (cells, extracellular matrix) are well described in the literature [128]. In the case of diabetes-complicated CRC and liver metastasis, results of a recent study indicate that the expression of biglycan is particularly intense in the myxomatous stroma. Induction of its production in vitro (HT29 cells) is regulated by high sugar concentration, fatty acids, and insulin. In turn, the co-culture with mesenchymal stem cells (MSCs) resulted in enhanced stemness and EMT phenotype [129]. 

In the case of CRC, in contrast to normal ECs, TECs originate not only from EPCs but also from the differentiation of CSCs. It was shown that CRC cells (HCT116 line) can transform into TECs under hypoxia conditions via a VEGFR-2-dependent mechanism. These cells expressed EC markers and formed tube-like structures in vitro [130]. Characterization of CRC blood supply and the role of TECs in this type of cancer, depending on its stage and immune remodeling, can both be found in a recent review [37]. Recent studies on tumor vascular ECs (referred as TVECs) purified from CRC tissues using iTRAQ-based quantitative proteomics analysis, among several groups of differentially expressed proteins (DEPs) and signaling pathways, also indicated proteins important in angiogenesis (e.g., HIF1 and PI3K/Akt signaling pathway-related proteins) were upregulated in TVECs compared with the controls [131]. The role of EPCs in CRC angiogenesis was also emphasized, with these cells exhibiting the potential to increase the tumorigenic capacity of CRC spheroid cells through angiogenesis, making them responsible for CRC progression [112]. 

## 7. Tissue Expression and Serum Levels of Wnt/β-Catenin Signaling Molecules–Diagnostic and Prognostic Role in CRC

When it comes to the expression of Wnt signaling components in CRC tissues, nuclear localization of β-catenin is described in the invasive front, in close proximity of the tumor microenvironment cells (known as the β-catenin paradox) [17,26,132,133]. Such localization mostly concerns isolated, scattered tumor cells [26]. Moreover, a correlation is described between nuclear β-catenin at the invasive front of the primary tumor and liver metastases [132,133]. Nuclear accumulation of β-catenin in neoplastic cells and the blood vessels was even considered as the most powerful predictor of liver metastasis in CRC [133]. However, there are also studies of rectal cancer, which did not detect any correlation between the nuclear overexpression of β-catenin and distant metastases or disease-free survival (DFS) [29]. Apart from its localization in the invasive front, a more heterogenous distribution of β-catenin can be observed intracellularly, both in cell membranes and in the cytoplasm [15,17,26,133,134]. Serafino et al. used a multiparametric analysis of IHC expression and subcellular localization of Wnt/β-catenin upstream (e.g., β-catenin, E-cadherin) and downstream signaling components (e.g., C-Myc, cyclin D1) in an animal model (rats) of chemically-induced CRC and human samples obtained from patients with inflammatory bowel diseases (IBD) or at sequential stages of sporadic CRC. A similar trend of β-catenin expression was noted in human and rat samples, reaching maximal values of nuclear β-catenin upregulation or membranous β-catenin downregulation in high grade dysplasia vs. normal mucosa. In advanced CRC from humans, membranous β-catenin was predominant vs. nuclear β-catenin. In their conclusions, the authors state that the crucial components of the Wnt pathway could be important markers for diagnosis, prevention, and therapy in IBD and sporadic CRC, and also possess a predictive value for responsiveness to Wnt-targeting therapy [134]. 

It was also noted that the cytoplasmic levels of β-catenin increased in response to hypoxia [60]. Dilek et al. showed nuclear expression of β-catenin in only 26.1% of rectosigmoid tumors, also reporting positive correlation between cytoplasmic β-catenin expression and VEGF [61]. The presence of the high Wnt signaling activity observed in tumor cells localized in the closest proximity to stromal myofibroblasts suggests a significant influence of the tumor microenvironment in further promotion of the nuclear translocation of β-catenin [15,16]. However, the prognostic role of nuclear β-catenin for distant metastases in rectal cancers is still a matter of discussion [29]. Nuclear localization of β-catenin at the invasive front of CRC appears to be important in early stages of colorectal carcinogenesis. However, there is not yet a consensus on the prognostic significance of such an expression pattern. It was stated that mutations in *APC* and *CTNNB*, while crucial for constitutive Wnt pathway activation, are not sufficient for nuclear β-catenin accumulation and full action of this signaling pathway [15,16]. 

Another protein of the Wnt pathway, increased expression of which in tumor cells is important for the initiation of inhibition of CRC angiogenesis process, is Wnt2 [108,120,121]. Positive expression of this protein was mainly demonstrated in stromal cells (CAFs), with little presence in cancer cells themselves. In turn, expression in CAFs positively correlates with clinicopathological data (depth of tumor, lymph node metastasis, TNM stage, venous invasion, and recurrence) [108]. Zhang et al. showed a significant positive correlation between tissue expression of Wnt2, collagen type VIII (COL8A1) (i.e., produced in ECs) and worse survival outcomes in CRC patients. Hence, Wnt2 and COL8A1 were deemed as independent factors of poor CRC prognosis. Moreover, high levels of Wnt2 expression were connected to ECM receptor and focal adhesion pathways [120]. Apart from higher β-catenin (mRNA, protein) in CRC tissues compared to the control, a correlation with elevated expression of CXCR4 was observed. Furthermore, a correlation between CXCR4 expression and low E-cadherin, high N-cadherin, and high vimentin was also noted, suggesting links between the SDF-1/CXCR4 pathway and Wnt/β-catenin signaling [84]. 

When it comes to the role of serum concentration of Wnt signaling components, as markers of CRC angiogenesis, it was proven that serum VE-cadherin was about fourfold higher in CRC patients compared with the controls, but it was not correlated with the VEGF level and any clinicopathological data (sex, age, tumor site, lymph node metastasis, grade, the subtype of CRC). Hence, the authors suggest that these proteins can be considered as independent markers of CRC angiogenesis [135]. 

## 8. Wnt/β-Catenin Signaling and Other Signalizing Partners in CRC Angiogenesis

The number of known Wnt/β-catenin signaling components and other pathways interacting with Wnt signaling, regulating angiogenesis, and enabling CRC progression continuously increases [35,66,68,86,106,136,137]. The pro-angiogenic pathways include Akt [71], PI3K/GSK3β [19], RAS-extracellular signal-regulated kinase (ERK) [20,138], PI3K/Akt/I kappa B kinase (IKK), PI3K/Akt/FOXO3a [2,136], PI3K/PTEN/Akt [68], cAMP/protein kinase A [137], SDF-1/CXCR4 [84], Norrin [115], Notch and VEGF-A/VEGFR-2 [127], miR-27a-3p/RXRα [139], ECM receptor, and focal adhesion [120] signaling pathways. 

In turn, anti-angiogenic signaling pathways interacting with Wnt signaling are TGF-β1 [77,98], HIF-1α/β-catenin/TCF3/LEF1 [98], and the protein kinase C-α (PKCα) signaling pathways [74,95,96,140]. 

## 9. The Role of Non-Coding RNAs in Angiogenesis via Wnt Signaling in CRC

MicroRNAs (miRNAs, MiRs) and long noncoding RNAs (lncRNAs) are two major families of non-protein-coding transcripts [141]. This group also includes circular RNAs (circRNAs), which are closed-loop RNAs formed by covalent bonds containing exons and introns [142]. The latter are generated via alternative back-splicing, which connects the terminal 5’ and 3’ ends of the single-stranded mRNA [143]. 

### 9.1. MicroRNAs (miRNAs, miRs)

MiRNAs are the most commonly studied form of non-coding RNAs, responsible for modulating up to 60% of protein-coding gene expression [144]. An increasing number of studies concerns the clarification of the role of micro-RNAs in CRC progression (including angiogenesis) via alteration of different signaling pathways including Wnt/β-catenin signaling (reviewed in [51,145]). 

Notably, increased expression of β-catenin in CRC tissues of mice (C57BL/6Apc(min/+) and human CRC cells positively correlated with significantly upregulated miR-574-5p. This miRNA changed the expression of β-catenin and p27 (Kip1 protein) as well as intensified the migration and invasion of cancer cells. Furthermore, in CRC tissues, miR-574-5p was negatively correlated with the expression of RNA binding protein Quaking (Qki) (associated with developmental defects in vascular tissues) [146]. 

Another study, among 26 deregulated miRNAs in an APC-inducible cell line, identified members of the miR-17-92 cluster that were inhibited by APC. In this process, the stabilized form of β-catenin (as a result of APC mutation) bound to and activated the miR-17-92 promoter. The main mechanism by which APC exerted its tumor suppressor activity was the reduction of miR-19a, the most important member of the miR-17-92 cluster. Therefore, the expression of miR-19a correlated with the level of β-catenin in the CRC samples, and was associated with an aggressive stage of cancer [147]. MiR-92a exhibits oncogene functions, being upregulated in chemoresistant CRC cells and tissues as well as intensifies Wnt/β-catenin signaling through Kruppel-like factor 4 (KLF4), GSK3β, and DKK-3. miR-92a expression was enhanced by IL-6/STAT, directly targeting its promoter. The authors also proved that increased miR-92a resulted in increased Wnt signaling and promotion of stem-like phenotypes of CRC cells [148]. 

Upregulation of miR-452 in ~70% CRC tissue samples vs. normal tissues was also reported, correlating with the clinical data. This MiR-452 promotes nuclear relocalization of β-catenin and the expression of target genes (e.g., C-Myc and cyclin D1). In turn, in vitro and xenograft mice models showed that MiR-452 can activate Wnt/β-catenin signaling and promotes an aggressive CRC phenotype through direct regulation of the 3’ untranslated region (3’UTR) of GSK3β. The miR-452 promoter is affected by the same transcription factors (TCF/LEF family of transcription factors). The authors conclude that a miR-452-GSK3β-TCF4/LEF1 positive feedback loop has an important role in CRC initiation and progression (including angiogenesis) [149]. 

Other MiRs promoting CRC proliferation, migration, invasion, and suppression of apoptosis in vitro, and in vivo include miR-27a-3p. This molecule acts through downregulation of nuclear receptor retinoid x receptor alpha (RXRα). On the tissue level, an increased expression of this MiR was demonstrated, correlating negatively with RXRα, and positively with various clinical (clinical-stage, distant metastasis, patients’ survival) and histological data (tumor differentiation). The authors also noted that RXRα negatively regulates the expression of β-catenin by its ubiquitination in CRC [138]. This confirms earlier observations of the aberrant expression of β-catenin, upregulated by suppression of RXRα [150] as well as direct interactions between RXRα and β-catenin, which suppress β-catenin transcription and protein expression in CRC cells [151]. 

MiR-224 [152] or epigenetic silencing of miR-490-3p [153] also promotes the aggressive CRC phenotype through activation of Wnt/β-catenin signaling. Direct regulative effects of MiR-224 on the 3’UTR of GSK3β and secreted Frizzled-related protein 2 (SFRP2) genes was demonstrated, leading to the activation of Wnt signaling and nuclear localization of β-catenin. Furthermore, ectopic miR-224 expression enhanced CRC proliferation and invasion [152]. 

On the other hand, miR-490-3p inhibits β-catenin and suppresses cell proliferation as well as lowers cell invasiveness by repressing EMT. Its direct target was identified as the protooncogene frequently rearranged in advanced T-cell lymphoma 1 (FRAT1) protein, which is linked with nuclear accumulation of β-catenin. Furthermore, hypermethylation of the miR-490-3p promoter downregulated the expression of this miR in CRC cells. The authors conclude that alterations in the miR-490-3p/FRAT1/β-catenin pathway can play an important role in CRC progression (including angiogenesis) [153]. 

Antagonistic action in transactivation of Wnt signaling is also exhibited by ectopic miR-29b expression. This miR acts through downregulation of β-catenin coactivators (TCF7L2, Snail, BCL9L) in colon cancer cells (SW480). It binds the 3’UTR of BCL9L, lowering its expression and reducing nuclear translocation of β-catenin. As a consequence, MiR-29b inhibits anchorage-independent cell growth, promotes EMT reversal, and reduces the ability of CRC cell-conditioned medium to induce in vitro tube formation in ECs [154]. 

### 9.2. Long-Non Coding RNAs (lncRNAs)

Other commonly investigated molecules taking part in different stages of CRC progression (including angiogenesis) also include long non-coding RNAs [31,155,156,157]. These conserved, small non-coding RNAs, made up from 21–25 nucleotides, act as negative regulators of gene expression. In the context of angiogenesis, they are also known as “angiomiRs”, directly or indirectly influencing this process (reviewed in [53]). Among this group of molecules, the Wnt/β-catenin signaling activating ability is attributed to lncRNA SLCO4A1-AS1. This molecule promotes β-catenin stabilization, impairing β-catenin-GSKβ interactions, and inhibiting its phosphorylation [156]. 

In turn, inhibition of tumorigenesis and progression (including angiogenesis and metastasis) in CRC is caused by lncRNA-CTD903 [155] and lncRNA-APC1 [158]. In CRC tissues, strong upregulation of CTD903 expression compared with adjacent normal tissues was observed. Furthermore, in the CTD903 knockdown model in CRC cell lines (RKO and SW480), both cell invasion and migration increased with EMT characteristics as well as reduced adherence ability. Downregulation of this lncRNA resulted in Wnt/β-catenin activation with increased transcription factors expression (e.g., Twist, Snail) [155], whereas overexpression of lncRNA-APC1 was sufficient to inhibit CRC cell growth, metastasis, and tumor angiogenesis by suppressing exosome production. Moreover, the results showed the oncogenic role of CRC-derived exosomal Wnt1, which acts in an autocrine manner through non-canonical Wnt signaling [158]. 

Inhibition of the Wnt signaling is also mediated by upregulation of lncRNA growth arrest specific 5 (lncRNA GAS5). This type of lncRNA plays a pivotal role in the prevention of angiogenesis, inhibiting invasion and CRC metastasis [31]. Other types of lncRNAs involved in Wnt signaling in CRC metastasis (e.g., colon cancer associated transcript 1/2 (CCAT-1/2), CASC11, PVT1, Wnt-regulated lincRNA-1 (WiNTRLINC1), PCAT1, and CCAL) are presented in recent reviews [157]. 

### 9.3. Circular RNAs (circRNAs)

One circRNA, namely circular decaprenyl-diphosphate synthase subunit 1 (PDSS1) was upregulated in CRC tissue compared to the control samples. All experiments showed that circPDSS1 is linked with local and distant metastasis as well as poor prognosis in CRC patients. Moreover, it was reported to stimulate angiogenesis in CRC via Wnt/ β-catenin signaling. Knockdown experiments resulted in attenuated migratory ability and angiogenesis in CRC cells. The authors noted a downregulation of Wnt/β-catenin signaling proteins including β-catenin, GSK3β, C-Myc, MMP-9, and cyclin D1 protein levels in CRC transfected with sh-cicrPDSS1 [159]. 

The main types of non-coding RNAs in CRC angiogenesis regulated by Wnt/β-catenin signaling-mediated mechanisms are summarized in Table 2.

## 10. Anti-Angiogenic Therapy in CRC

Treatment of CRC patients, especially those affected by metastatic CRC (mCRC), still poses a major challenge and requires significant treatment personalization. Different forms of anti-angiogenic therapy have been attempted, taking into account the mechanisms of CRC angiogenesis, in which a major role is played by the VEGF pathway. There have been approaches based on the application of anti-angiogenic small-molecule TKIs (e.g., sorafenib, sunitinib, vatalanib, or tivozanib), with or without chemotherapy. Furthermore, monoclonal antibodies have also been used, both anti-VEGF pathway and EGFR targeting (cetuximab and panitumumab). The effectiveness of typical anti-VEGF-R TKIs (regorafenib, famitinib, axitinib, and apatinib) turned out to greatly vary in mCRC treatment. The first, most effective multikinase inhibitor of angiogenic (including VEGFR-1, -2, -3), stromal and oncogenic receptor TK, was regorafenib [160,161,162]. This drug evoked the most significant effects in cases of advanced, refractory disease [161], especially in anti-angiogenic-naïve patients with chemotherapy-refractory mCRC. The therapy with regorafenib showed antitumor activity in 59 CRC patients in a single-center, single-arm phase IIb study [162]. The most recent open-label, single center, single-arm, phase 3 study indicates clinical effectiveness of another multikinase inhibitor, lanvatinib, in the therapy of unresectable mCRC patients, especially refractory or intolerant to classical chemotherapy, anti-VEGF therapy, and anti-EGFR therapy (tumor with wt-*RAS* expression) [163]. Furthermore, promising results have also been reported for another highly-selective anti-VEGFR-1, -2, and -3 small molecule, fruquintinib, which improved both overall survival (OS), and progression-free survival (PFS) in mCRA patients compared with the placebo. This TKI was approved by the China Food and Drug Administration (CFDA) (2018) for mCRC patients after at least two standard anticancer therapies [164]. 

However, anti-angiogenic CRC therapies (also those combined with other forms of treatment) are not fully effective, being a matter of discussion in many excellent reviews [36,160,161,165]. Many individual variations have been observed in response to anti-angiogenic factor therapies, sparking the search for new compounds and/or identification of susceptibility markers [160,161,166]. An analysis of the profile of the expression of genes important for an effective response to cetuximab (anti-EGFR-targeted agent) therapy in 80 CRC tumors allowed for the identification of six clinically relevant CRC subtypes. Each of those subtypes showed differing degrees of “stemness” and Wnt signaling [32]. Furthermore, there has been a perspective for the improvement of efficacy and more targeted treatment in the form of studies on host genetic markers (reviewed in [166]). 

There are currently a few anti-angiogenic agents approved by the U.S. FDA for mCRC treatment: anti-VEGF/VEGF-R agents (e.g., bevacizumab, ziv-aflibercept, regorafenib, ramucirumab), anti-EGFR agents (e.g., cetuximab, panitumubab), or immune-check-point inhibitors (e.g., pemprolizumab, nivolumab, ipilimubab). However, bevacizumab is the only anti-angiogenic compound for the first-line treatment of mCRC (from 2004) [36,165]. The subgroup analysis from the CONCUR trial suggests that regorafenib treatment prior to targeted therapy (including bevacizumab) may improve clinical outcomes [162]. 

### Wnt/β-Catenin Signaling as a Potent Therapeutic Target in CRC-Associated Angiogenesis

Apart from anti-angiogenic therapy based on the VEGF pathway, Wnt/β-catenin signaling is among the pathways offering potential sites for targeting [140,165,167,168]. Most studies aiming to establish the most efficient anti-Wnt/β-catenin therapy concerned a better understanding of the mechanisms regulating APC signaling and/or factors downstream of APC that control β-catenin stability and/or co-transcriptional activity [74,140,168,169]. The confirmed factors inhibiting the Wnt/β-catenin signaling pathway include examples of potential CRC therapeutic factors, most of them exhibiting anti-tumor activity [83]. Their effects are mainly exerted through the inhibition of cell proliferation/migration/invasion, cancer progression delay as well as the prevention of CRC metastasis [83,140,165]. Furthermore, some drugs can be used to eliminate chemotherapy-resistance [167]. 

The most commonly mentioned existing anti-angiogenic drugs targeting the Wnt/β-catenin pathway in CRC include non-steroidal anti-inflammatory drugs (NSAIDs) (e.g., sulindac and celecoxib), which can “bypass” many carcinogenic effects, also regulating the increased expression of PTEN and GSK3β, inhibition of Akt (and β-catenin), and MMPs as well as iNOS activation, all of which induce cancer cell apoptosis [68,167]. Other anti-inflammatory drugs (e.g., artesunate and aspirin) caused a marked reduction in preneoplastic changes in a rat model. Both drugs also downregulated Wnt/β-catenin signaling and reduced the levels of angiogenic markers like VEGF and MMP-9. These drugs inhibited cellular proliferation and resulted in pro-apoptotic effects [170]. 

Apart from NSAIDs, vitamin A and D derivatives also showed efficacy in the disruption of a number of signaling pathways (e.g., Erk and PI3K/Akt) including Wnt. This fact, together with the introduction of new generations of their derivatives, creates a perspective for potential new interesting clinical trials [171,172,173,174]. Vitamin D3 metabolites, which generally inhibit growth and induce differentiation of cancer cells, have been found to also exert anti-proliferative effects on CRC cell lines (LoVo, HT29, and HCT116) and clinical samples [171]. There are reports stating that the active form of vitamin D3 and its analogs inhibit proliferation, angiogenesis, migration/invasion, and induce differentiation and apoptosis in malignant cell lines including CRC cells (reviewed in [175]). One of the newfound anti-tumor effects of 1,25(OH)_2_D_3_ in human CRC occurs through the DKK-1 gene induction [176] and DKK-4 gene downregulation, both considered as novel mechanisms of Wnt signaling inhibition [81]. The use of protein-vitamin D-pectin nano-emulsion (NVD) induces cytotoxicity in CRC cells in a dose- and time-dependent manner. This compound inhibits the growth of CRC cells (HCT116 and HT29) through the regulation of proteins responsible for the G2 phase of the cell cycle (cyclins A, B1, E2, and decrease in Cdc25c) as well as encourages apoptosis. In the context of Wnt/β-catenin signaling, NVD causes a decrease in expression of β-catenin (mRNA, protein), Akt, and survivin genes in vitro as well as in vivo (mice xenograft model). NVD administration in CRC cells decreases PI3K and Akt phosphorylation as well as inhibits β-catenin production. Hence, the inhibitory effects of vitamin D derivatives on CRC cells also depend on blocking the Wnt/β-catenin signaling and its downstream targets (e.g., survivin) [173]. Therefore, NVD, as a Wnt/β-catenin inhibitor, has the potential to stop tumor invasion and metastasis processes (including angiogenesis). It was also proven that calcitriol (1α,25-dihydroxyvitamin D3), as an active vitamin D metabolite, inhibits the tumor-promoting properties of patient-derived CAFs, also modulating many types of immune cells expressing vitamin D receptor (VDR) [177]. Other mechanisms and factors increasing the anti-proliferative action of vitamin D derivatives in CRC cells (SW480) have also been examined. These include cytochrome P450 family 24 subfamily A member 1 (CYP24A1), overexpression of which can be observed in CRC. It was recently proven that CYP24A1 inhibition induces translocation of β-catenin from the nucleus to the cell membrane in SW480 cells, intensifying the inhibitory effect of 1,25(OH)_2_D_3_ on C-Myc. Methylation of this factor increased the anti-tumor effects of vitamin D in CRC [172]. 

In turn, when it comes to vitamin A and its derivatives, it is worth noting that the pathways directing β-catenin for proteasome degradation (in addition to p53/Siah-1/APC and Wnt/GSK3β/APC) include the RXR-mediated pathway [178,179]. It was proven that retinol decreases the levels of β-catenin and increases ubiquitinated protein in three all-trans retinoic acid (ATRA)-resistant human CRC cells (HCT-116, WiDr, and SW620). Retinol treatment lowered the transcription of the TOPFlash reporter and mRNA levels of the endogenous β-catenin target genes (cyclin D1 and C-Myc). Hence, the potential influence of retinol on colon cancer cell growth inhibition occurs through an increase in β-catenin degradation in proteasomes with the use of the RXR-mediated pathway [180]. The research of the same group confirmed that retinol administration to ATRA-resistant human CRC cells increased β-catenin and RXRα protein interactions, inducing β-catenin transport to the degradation location in the cytoplasm [179]. 

A growing number of novel agents targeting the Wnt pathway are subjected to clinical trials including specific small molecules [165,168]. This group includes G007-LK and G244-LM, specific tankyrase inhibitor compounds, which reduce Wnt/β-catenin signaling through the prevention of Axin degradation, resulting in the promotion of β-catenin destabilization [169]. As β-catenin is considered the primary cause of dysregulated Wnt signaling, the action of a range of its direct inhibitors and knockdown strategies was examined. However, as of today, none of them have been introduced into oncological practice [74,140]. The usefulness of current approaches to target anti-Wnt therapy against CRC is the subject of recent reviews [181]. Furthermore, ongoing clinical trials 1-2 employ novel agents affecting this signaling pathway (with Wnt as targets), for example, Wnt-974, Foxy-5, and LGK-974 [165,167]. 

It was proven that aberrant activation of Wnt/β-catenin signaling mediates resistance of CRC cells to irradiation and 5-FU-based chemotherapy. Higher levels of active β-catenin and increased TCF/LEF reporter activity were observed in SW1463 cells that evolved radiation resistance. It was also demonstrated that inhibition of β-catenin (via siRNAs or small-molecule inhibitor of β-catenin transcription, XAV-939), sensitized CRC cells to chemoradiotherapy [182]. Other studies in in vitro and mice xenograft tumor models showed that PI3K/Akt signaling inhibition leads to nuclear β-catenin and FOXO3a accumulation (both promoting metastasis). It was proven that nuclear β-catenin confers resistance to the FOXO3a-mediated apoptosis induced by PI3K and Akt inhibitors (API-2), with this effect reversed by XAV-939 [2]. 

Recently, previous observations on some Wnt/β-catenin signaling inhibitors and downstream targets involving PKCα came back to light, indirectly related to the progression of CRC and angiogenesis. It was proven that PKCα is rarely mutated in CRC samples, hence its function might be activated with no side effects for the intestinal epithelium. Additionally, PKCα activation results in increased cell death and is drug-inducible. According to the authors of the study, there are ongoing phase II clinical trials on the application of natural PKCα activators (found in the Bryozoan species *Bugula neritina*) for CRC treatment [74]. The use of a stabilized form of BCL9 α-helix (SAH-BCL9) is also suggested in potential therapy, as its administration caused dissociation of the native β-catenin/BCL9 complex as well as suppressed tumor growth and angiogenesis in the mouse xenograft model of the Colo320 CRC cell [83]. 

Moreover, a growing number of publications have documented the action of anti-Wnt/β-catenin signaling plant based compounds (particularly those used in traditional Chinese medicine) However, anti-angiogenic actions linked to Wnt signaling are only attributed to some of them, for example, Raddeanin [103,104,105] and Tanshinone IIA [39,98,183] (Table 1). Other naturally occurring compounds that inhibit Wnt signaling include thymol, derived from *Thymus vulgaris L* [184,185]. One of the mechanisms of this factor’s action in CRC in vitro (HCT116 and LoVo cells) as well as in vivo is the prevention of EMT, invasion, and metastasis through the inhibition of Wnt/β-catenin signaling [186]. In turn, in the case of Radix *Tetrastigma hemsleyani* flavone (RTHF), it was proven that this compound causes downregulation of β-catenin activation and downstream protein expression (Lgr5, C-Myc, and cyclin D1). It also decreased the size of tumors in vivo in mice through the inhibition of pro-proliferative properties of the Wnt pathway [187]. Another plant-based polyphenol compound extracted from the root of *Curcuma longa*, is curcumin. This phytochemical also shows an anti-inflammatory, anti-oxidant, and anti-cancer activity [188]. In studies of CRC cells (SW480) as well as in the xenograft tumor model, Dou et al. proved anti-tumor activity of curcumin via inhibition of cell proliferation by suppression of the Wnt/β-catenin pathway. It was also noted that overexpression of miR-130a could abolish the anti-tumor activity of curcumin [189]. Recently, a study of another CRC cell line (SW620 cells) reported an inhibitory influence of curcumin on cell viability as well as the promotion of apoptosis. At the same time, an increase in the expression of Caudal Type Homeobox-2 (CDX2) and decreased β-catenin nuclear translocation were observed. In turn, the expression of downstream proteins of Wnt/β-catenin signaling (Wnt3a, C-Myc, survivin, and cyclin D1) was reduced. Furthermore, it was reported that the inhibitory action of Wnt/β-catenin in these cells occurred due to CDX2 restoration [190]. The isobavachalcone, a flavonoid extracted from *Psoralea corylifolia*, also inhibits growth and colony formation of CRC tumor cells as well as the induction of apoptosis through the inhibition of the AKT/GSK3β/β-catenin pathway have been noted [191]. Promising study results in anti-Wnt/β-catenin signaling therapies also concern berberine (and its synthetic 13-arylalkyl derivatives) [192,193], an isoquinoline alkaloid present in several plants including *Coptis sp.* and *Berberis sp.* [194]. Special attention was given to its anti-tumor function, mediated by the inhibition of β-catenin transcriptional activity and weakening of anchorage-independent growth (decrease in E-cadherin expression) [192]. It was proven that berberine inhibits the function of β-catenin by direct binding to a unique RXRα region that contains the Gln^275^, Arg^316^, and Arg^371^ residues. As a result, a promotion of this receptor’s interaction with nuclear β-catenin occurs, leading to c-Cbl mediated degradation of β-catenin, hence the inhibition of cell proliferation. Moreover, human CRC xenograft in nude mice also demonstrated the inhibition of tumor growth in an RXRα-dependent manner [193]. 

The basic drawbacks of anti-angiogenic and anti-Wnt signaling targeted therapies have been presented in several reviews [54,165]. These include the costs of treatment, extra adverse events, crossover, and bypass mechanisms between different signaling pathways and drug resistance as well as varying efficacy among patients [165]. The main challenges and complexities associated with creating the perfect therapeutic agents targeting the Wnt/β-catenin signaling pathway in CRC have been summarized by others [140]. 

Direct CRC angiogenesis inhibition mechanisms based on Wnt/β-catenin signaling are only described in a small number of existing or potential therapeutics. Nevertheless, previously mentioned results of studies (Section 2, Section 3 and Section 4) demonstrate a tight interaction of Wnt signaling with angiogenesis markers in CRC. It can therefore be assumed that the inhibition of upstream and/or downstream targets of Wnt signaling, apart from downregulating cell proliferation/migration/invasion, hence tumor growth and metastasis, is also a statement of angiogenesis inhibition in the tumor. More detailed information on the therapeutics targeting the Wnt/β-catenin signaling pathway in CRC can be found in existing works focused solely on this topic [140]. Table 3 summarizes the selected existing drugs and several agents under investigation for different Wnt/β-catenin targets in CRC with an indication of their influence on angiogenesis.

## 11. Final Remarks and Future Perspectives

Angiogenesis belongs to the most clinical characteristics of CRC and is strongly linked to the activation of Wnt/β-catenin signaling. The most prominent factors stimulating constitutive activation of this pathway, and in consequence angiogenesis, are genetic alterations (mainly mutations) concerning *APC* and the β-catenin encoding gene (*CTNNB1*), detected in a large majority of CRC patients. These mutations lead to an intensification of CRC cell proliferation, migration, and invasion in vitro as well as tumor growth, angiogenesis, and distant metastases in vivo. In addition to the mutations mentioned, there are more and more genetic and epigenetic biomarkers used to determine CRC diagnosis, prognosis, and response to therapy, as summarized in excellent reviews [202]. There are also potential clinical applications of liquid biopsy biomarkers in CRC including circulating tumor cells, circulating tumor DNA, miRNAs, lncRNAs, and proteins from blood and body fluids, and their genomic and proteomic analyses (reviewed in [203]).

Wnt/β-catenin signaling is involved in the basic types of vascularization (sprouting and nonsprouting angiogenesis) and vasculogenic mimicry as well as the formation of mosaic vessels. In vascular cells, expression of Wnt ligands, Wnt receptors, and Wnt inhibitors has been reported. The main type of angiogenesis with the participation of Wnt signaling is currently assumed to occur through the hypoxia-adaptation mechanism mediated by VEGF-signaling and upregulation of the HIF-1 complex. β-catenin itself induces the expression of VEGF in colon cancer cells in the early steps of CRC neoangiogenesis. Furthermore, tissue VEGF expression positively correlates with the cytoplasmic expression of β-catenin in tumor cells and tumor progression in vivo. In turn, the influence of HIF-1α (increasing) and HIF-2α (decreasing) on β-catenin levels/transcriptional activity in CRC cells remains much more varied. Moreover, non-endothelial interactions between both VEGF receptor types (VEGFR-1, VEGFR-2) and Wnt/β-catenin signaling have also been reported. It was confirmed that VEGFR-1 positively regulates Wnt signaling in a GSK3β-independent manner. In contrary to the previous paradigm, the presence of both VEGF receptor types was also demonstrated on tumor CRC cells, suggesting the possibility of autocrine VEGF action. 

Factors regulating angiogenesis with the participation of Wnt/β-catenin signaling include different groups of biologically active molecules, namely selected molecules belonging to Wnt family proteins (e.g., Wnt2, DKK, BCL9) as well as various factors outside the Wnt family (e.g., DHX32, gankyrin, Uba2, CXCL8, SALL4, FOXQ1, bioactive compounds of plants, etc.). 

A direct influence of several pro-angiogenic factors (e.g., BCL9, SALL4) on Wnt signaling has been demonstrated (binding β-catenin) in the angiogenesis process. Other factors promoting angiogenesis (e.g., DHX32, gankyrin, Uba2, AKT) regulate Wnt signaling through β-catenin stabilization and increase Wnt gene expression as well as the intensification of EMT-related transcription factor expression (including β-catenin). This regulation results in EC migration and the formation of capillary-like tubules of human microvascular ECs. The opposite effects are evoked by the anti-angiogenic factors through the inhibition of production and transcriptional activity of β-catenin (e.g., TIPE2, SMAR1, PKG, PKCα, sporamin, emodin, 6-Gingerol, raddeanin A). Recently, an increasingly important role in Wnt signaling involving CRC angiogenesis is attributed to non-coding RNAs. A number of these molecules activate (e.g., miR-574-5p, miR-17-92, miR-92a, miR-452, miR-27a-3p, miR-224, lncRNA SLCO4A1-AS1, and circPDSS1), while other inhibit Wnt signaling (e.g., miR-490-3p, miR-29b, lncRNA-CTD903, lncRNA APC1, and lncRNAGAS5). 

The active cellular components of CRC-related angiogenesis consist of tumor cells, CRC stem cells, and cancer-associated fibroblasts (CAFs) as well as cells directly linked to blood vessels (EPCs, TECs, pericytes). Moreover, complex intercellular interactions have been reported in tumors during angiogenesis. CRC cells produce β-catenin (mRNA and protein), which intensifies VEGF expression and increases vessel density. The norrin protein produced by cancer cells binds to Fzd4, regulating EC proliferation and motility. In turn, norrin/Fzd4 interactions are modulated via regulation of Fzd4 expression by Wnt2. Furthermore, exosomes enriched in Wnt4 produced by CRC cancer cells promote angiogenesis by increasing ECs proliferation and migration via Wnt signaling. Both tumor CRC cells and CAFs (main source) produce the Wnt2 protein, which plays a major role in the initiation and maintenance of the CRC angiogenesis process. Wnt2 expression in CAFs correlates with a number of clinicopathological data (including venous invasion) of CRC patients. Wnt2 intensifies EC migration and invasion, enhanced vessel density, and tumor volume. Wnt2 expression positively correlates with the expression of vascular markers and an increase in pro-angiogenic function of many proteins (e.g., IL-6, G-CSF, and PGF). When it comes to CRC stem cells, high Wnt activity is mostly present in the bottom third of the crypts (where CSCs reside). These cells have the ability of transdifferentiation into human TECs as well as the generation of functional blood vessels. 

The list of Wnt/β-catenin signaling components and pathways interacting with Wnt signaling, regulating angiogenesis, and conditioning CRC progression, continuously increases. 

As β-catenin is considered as a primary cause of dysregulated Wnt signaling in CRC as a consequence of APC/*CTNNB1* mutations, there are ongoing studies on the action of a number of inhibitors of β-catenin itself as well as knockdown strategies. However, no results of such research have yet been introduced into CRC oncological practice, due to the relatively low effectiveness as well as significant intestinal toxicity. Small molecules blocking Wnt signaling in CRC also include tankyrase inhibitors (G007-LK and G244-LM). There are several clinical trials (phase 1/2, phase 1, and phase 2) on the use of novel Wnt targeting agents in CRC (e.g., Wnt-974, LGK-974, Foxy-5). Positive anti-angiogenic effects, disrupting Wnt/β-catenin signaling have been demonstrated for a number of NSAIDs (e.g., sulindac, celecoxib, artesunate, and aspirin) and vitamin A and D derivatives. Furthermore, many natural plant-derived compounds used in traditional Chinese medicine inhibits Wnt/β-catenin signaling and, directly or indirectly, CRC angiogenesis (e.g., RA, thymol, RTHF, curcumin, IBC, Tan IIA, and berberine). As for now, the available results mostly concern in vitro and mouse in vivo models. 

## 12. Conclusions

As the reviewed literature shows, the role of aberrant Wnt/β-catenin signaling in CRC-related angiogenesis is undisputed. These activities mostly occur due to canonical APC/β-catenin pathway activation in tumor colorectal cells, CRC stem cells, cancer-associated fibroblasts and tumor ECs, intensification of β-catenin expression, and translocation to the nucleus as well as positive correlations with other typical pro-angiogenic factors (e.g., VEGF, VEGRs). Furthermore, the role of a number of active polypeptides, proteins, and non-coding RNAs is indicated in this process. However, when it comes to anti-angiogenic CRC treatments based on targeting the Wnt/β-catenin signaling, studied inhibitors of this pathway are still mostly in preclinical stages, with only a few compounds reaching phase 1 or 2 clinical trials. Individualized targeted CRC therapeutic strategies should take into account the newest findings of molecular biology, explaining the role of direct tumor cell interactions, and all pro- and anti-angiogenic factors acting on this type of signaling as well as other related pathways. An especially large number of publications in the last five years focusing on the role of Wnt/β-catenin signaling in cancer progression (including CRC) has certainly resulted in a better understanding of the mechanisms of metastasis as well as improvements in the management of this cancer. 

## Figures and Tables

**Table 1:** A list of known pro- and anti-angiogenetic factors and their influence on the regulation of the Wnt/β-catenin signaling pathway in colorectal cancer (CRC) angiogenesis.

Action	Family of Factors	Factor	Molecular Mechanisms/Effects on Angiogenesis	Ref.
Pro-angiogenic	Wnt pathway components	DKK-4	(i) ↑expression in CRC cells; (ii) ↑migration and formation of capillary-like tubules of human primary microvascular ECs	[81]
		BCL9	(i) directly binds to β-catenin; (ii)BCL9 knockdown attenuated the expression of pro-angiogenic factors (e.g., CD44, and VEGF), which resulted in a reduction of tumor metastasis and angiogenesis	[67,79,80]
		Wnt2	(i) ↑expression in CAFs, which correlates with clinical data; (ii) induces CRC cells and EC migration and invasion; (iii) ↑vessels density and tumor volume; (iv) activates Wnt signaling in autocrine and paracrine manner	[106,107,108]
	Non-Wnt pathway factors	DHX32	(i) ↑VEGF-A and stabilization of β-catenin; (ii) ↑↑- is a poor prognostic factor	[72]
		gankyrin (PSMD10)	(i) coordinates cooperation between PI3K/Akt and canonical Wnt/β-catenin signaling pathways; (ii) overexpressing gankyrin promoted angiogenesis, chemoresistance and metastasis of CRC cells both in vitro and in vivo	[19]
		Uba2	Regulates Wnt signaling and enhances EMT	[85]
		TGM2	↑expression of MMP-2, MMP-9, Wnt3a, β-catenin and cyclin D1	[75,86]
		c-Cbl gene	Mutant *C-Cbl-Y371H* shows ↑Wnt/β-catenin signaling, increased Wnt genes, angiogenesis, and CRC growth via phosphorylation of c-Cbl Tyr^371^	[73]
		AKT	↑↑EMT-related transcription factors (including β-catenin)	[71]
		CXCL8	(i) ↑VEGF-A and Bcl2; (ii) ↑cell invasion via AKT/GSK3β/β-catenin/MMP7 pathway	[70]
		CXCR4	SDF-1 induces CXCR4-positive CRC cell invasion and EMT via activation of Wnt/β-catenin signaling	[84]
		SALL4	(i) directly binds to β-catenin; (ii) co-expression with β-catenin promoting lymph node metastasis and advanced stage; (iii) modulates VEGF-A expression in HUVECs	[76,78,87]
		FOXQ1	(i) ↑↑correlates with stage and lymph nodes metastasis; (ii) modulates cell invasion, EMT, and resistance to drug-induced apoptosis	[77]
	Bioactive compound of plants	Aloin, aloesin	(i) aloin activates Wnt/β-catenin signaling in the presence of Wnt3a in CRC cells; (ii) aloesin directly activates Wnt signaling in Wnt3a independent manner	[89]
Anti-angiogenic	Wnt pathway components	DKK-1	(i) ↓MVD and VEGF expression vs. control; (ii) inhibits tube-like structure formation and ↓VEGF expression in HUVECs	[90]
	Non-Wnt pathway factors	TIPE2 (TNFAIP8L2)	↓expression of Wnt3a, phospho-β-catenin, and GSK-3β in rectal adenocarcinoma cells; (ii) cooperates with Wnt/β-catenin and TGF-β/Smad2/3 signaling pathways	[91]
		SMAR1	Inhibits β-catenin mRNA production and promoter activity by recruiting Histone deacetylase-5 to β-catenin promoter	[94]
		PKG	↓VEGF and β-catenin expression in TCF-dependent transcription	[93]
		PKCα	(i) inhibits β-catenin transcriptional activity via Wnt5a/PKCα-dependent phosphorylation on SER35 of ROR α; (ii) phosphorylates of β-catenin	[95,96]
	Bioactive compound of plants	Aloin	(i) inhibits HUVECs proliferation, migration and tube formation in vitro; (ii) inhibits VEGFR-2 and STAT3 phosphorylation in ECs; (iii) ↓VEGF antiapoptotic, pro-proliferative factors (C-Myc) in CRC cells	[88]
		Sporamin	↓β-catenin and VEGF production	[97]
		Tan IIA (TSA)	(i) inhibits secretion of VEGF and bFGF; (ii) suppresses the proliferation, tube formation and metastasis of HUVECs; (iii) inhibits β-catenin/VEGF-mediated angiogenesis by decreasing TGF-β (via HIF-1α inhibition)	[98]
		Emodin	(i) ↓TCF/LEF transcriptional activity; (ii) inhibits EMT proteins, β-catenin and TCF7L2, VEGF production; (iii)↑cadherin E mRNA expression	[99,100]
		6-Gingerol	(i) inhibits Wnt3a and β-catenin expression; (ii) ↓VEGF, ANG-1, FGF, GDF-15 levels	[101]
		Raddeanin A	(i) modulates VEGF-mediated phosphorylation of VEGFR-2 and downstream kinases FAK, PLCγ1, Src, and Akt; (ii) inhibits p-LPR6, inactivates AKT, removes GSK-3β inhibition and attenuation of β-catenin; (iii) inhibits HUVECs proliferation, motility, migration, and tube formation	[104,105]

Abbreviations: ↑,↓—increase (upregulation)/decrease expression/level; ↑↑—overexpression; AKT (Akt)—Protein Kinase B; ANG-1—Angiopoietin-1; Bcl-2—B-cell lymphoma protein; BCL9—B cell lymphoma 9; CAFs—Cancer Associated Fibroblasts; c-Cbl—Casitas B-lineage lymphoma gene; CXCL8—the chemokine (C-C motif) ligand 8; CXCR4—C-X-C chemokine receptor type 4; DHX32—DEAH box protein 32; DKK—Dickkopf-related Protein; ECs—Endothelial Cells; EMT—Epithelial-Mesenchymal Transition; FAK—Focal Adhesion Kinase; FGF—Fibroblast Growth Factor; FOXQ1—Forkhead Box Q1 Protein; GDF-15—Growth Differentiation Factor 15; GSK-3β—Glycogen Synthase Kinase 3 β; HIF-1α—Hypoxia-inducible Factor 1 α; HUVECs—Human Umbilical Vein ECs; LEF—Lymphoid Enhancer Factor; MMP-2, -9—Matrix Metalloproteinase 2, -9; MVD—Microvessel Density; PI3K—Phosphatidylinositol 3’ Kinase; PKCα—Protein Kinase C α; PKG—type 1 cyclic guanosine monophosphate (cGMP)-dependent protein kinase; PLCγ1—Phospholipase C γ1; p-LPR6—phosphorylated Lipoprotein-related Protein 6; ROR α—RAR-related orphan receptor α; SALL4—Zink Finger Transcription Factor Spalt (Sall)-like Protein 4; SDF-1—Stromal Cell-derived Factor 1; SER—Serine; SMAR1—Scaffold/Matrix Attachment Region Binding protein 1; STAT3—Signal Transducer and Activator of Transcription Protein 3; Tan IIA/TSA—Tanshinone IIA; TCF—T cell Factor; TCF7L2—Transcription Factor 7-like 2; TGF-β—Tumor Growth Factor beta; TGM2—Tissue Transglutaminase 2; TIPE2 (TNFAIP8L2)—Tumor Necrosis Factor α (TNFα)-induced protein 8 like 2; VEGF (R)—Vascular Endothelial Growth Factor (Receptor).

**Table 2:** The role of selected non-coding RNAs in colorectal cancer (CRC) angiogenesis regulated by Wnt/β-catenin signaling-mediated mechanisms.

Type of Non-Coding RNAs		Interacting Molecules	Molecular Mechanism of Angiogenesis	Effect on Wnt Pathway	Ref.
miRNAs	miR-574-5p	Qki	(i) ↑expression correlated with ↑expression of β-catenin and p27 (Kip1 protein), cell proliferation, invasion, and migration; (ii) ↑expression inversely correlated with Qkis isoforms	activates	[146]
	miR-17-92 cluster (including miR-19a)	β-catenin	(i) β-catenin binds to and activates the miR-17-92 promoter; (ii) miR-19a correlates with β-catenin level and aggressive stage of CRC	activates	[147]
	miR-92a	Wnt/β-catenin	(i) ↑expression in CRC cells; (ii) enhances Wnt/β-catenin signaling through KLF4, GSK3β and DKK-3; (iii) increased miR-92a promotes of stem-like phenotypes of CRC cells	activates	[148]
	miR-452	3’-UTR of GSK3β; β-catenin	(i) ↑expression in ~70% CRC tissue and CRC cell lines; (ii) promotes nuclear relocalization of β-catenin and the expression of the target genes; (iii) direct regulation on the 3’-UTR of the GSK3	activates	[149]
	miR-27a-3p	RXRα	(i) ↑expression in CRC tissue and positive correlation with clinical data; (ii) negative correlation with RXRα; (iii) downregulation of RXRα which prevents β-catenin degradation	activates	[139]
	miR-224	3’-UTR of GSK3β and SFRP2 genes	(i) leads to nuclear translocation of β-catenin; (ii) upregulated miR-224 inhibits the expression of GSK3β/SFRP2 and enhances CRC proliferation and invasion	activates	[152]
	miR-490-3p	FRAT1	(i) ↓expression in CRC cells via hypermethylation of the miR-490-3p promoter; (ii) suppresses CRC cells proliferation, inhibits invasion (via repressing EMT); (iii) inhibits β-catenin expression in nuclear fractions of CRC cells	inhibits	[153]
	miR-29b	3’UTR of BCL9L	(i) downregulates coactivators of β-catenin (TCF7L2, Snail, BCL9L); (ii) decreases nuclear translocation of β-catenin; (iii) ↓tube formation in ECs	inhibits	[154]
lncRNAs	lncRNA SLCO4A1-AS1	Wnt/β-catenin	(i) ↑expression in CRC tissues correlates with poor prognosis and metastasis; (ii) promotes cell proliferation, migration, and invasion (via EMT); (iii) enhances β-catenin stability	activates	[156]
	lncRNA-CTD903	Wnt/β-catenin	(i) ↑expression in CRC tissues *vs.* control; (ii) is independent factor of favorable prognosis; (iii) downregulated enhances Wnt/β-catenin activation and their downstream transcription factors	inhibits	[155]
	lncRNA GAS5	Wnt/β-catenin	(i) weak expression in CRC tissues and cells; (ii) upregulated inhibits CRC cells invasion and migration in vitro; (iii) inhibits of tumor growth, angiogenesis, and liver metastasis in vivo	inhibits	[31]
	lncRNA-APC1	APC	(i) ↑expression inhibits CRC cell growth, metastasis, and tumor angiogenesis by suppressing exosome production; (ii) inhibits MAPK pathway in ECs and suppress angiogenesis	inhibits	[158]
circRNAs	circPDSS1	Wnt/β-catenin	(i) ↑expression in CRC tissues vs. control; (ii) higher level predicts high rates of metastasis, and overall survival; (iii) knockdown of *PDSS1* results in attenuation of migratory abilities and angiogenesis in CRC cells	activates	[159]

Abbreviations: ↑,↓—increase/decrease expression/level; APC—Adenomatous Polyposis Coli gene; BCL9L—B cell lymphoma 9-like protein; circ PDSS1—circular Decaprenyl-Diphosphate Synthase Subunit 1; circRNAs—circular RNAs; DKK—Dickkopf-related Protein; ECs—Endothelial Cells; EMT—Epithelial-Mesenchymal Transition; FRAT1—Frequently Rearranged in Advanced T-cell Lymphoma 1 Protein; GAS5—Growth Arrest Specific 5; GSK3β—Glycogen Synthase Kinase 3 β; KLF4—Kruppel-like Factor 4; lncRNA—long non-coding RNA; MAPK—A Mitogen-activated Protein Kinase; miR (miRNA)—microRNA; Qki—RNA family protein Quaking; RXRα—Nuclear Receptor Retinoid X Receptor alpha; SFRP2—Secreted Frizzled-related Protein 2; TCF7L2—Transcription Factor 7-like 2; 3’UTR—3’ Untranslated region of gene.

**Table 3:** Selected classes of existing/potential anti-Wnt/β-catenin signaling therapeutics with anti-angiogenic effects in colorectal cancer (CRC).

Class of Agents	Name of Targeted Agents	Target	Mechanism of Action and Effects in CRC Cells	Effect on Angiogenesis	Stage of Development	Ref.
NSAIDs	Sulindac (Clinoril)	β-catenin	(i) both drugs ↑ expression of PTEN and GSK3β, inhibit of Akt (and β-catenin), MMPs, and iNOS activation; (ii) inhibit proliferation, have pro-apoptotic effects; (iii) ↓CD133 expression, a marker of cancer stem cells; (iv) inhibit COX-2 and progression of tumor	inhibits	clinical	[68,140,167,195]
	Celecoxib	TCF		inhibits	clinical	
Other anti-inflammatory drugs	Artesunate	β-catenin	(i) both drugs down-regulate β-catenin signaling and ↓levels of VEGF, and MMP-9; (ii) inhibit proliferation, and have pro-apoptotic effects	inhibits	clinical	[170]
	Aspirin	β-catenin		inhibits	clinical	
Vitamins and their derivatives	Vitamin D3 metabolites (Cholecalciferol)	Wnt/β-catenin with upstream and downstream targets	(i) anti-proliferative effects in vitro and in vivo; (ii) ↑DKK-1 gene and ↓DKK-4 gene	inhibits	Phase 1–3 *	[81,140,171,172,173,174,175,176]
	Vitamin A and its other forms (e.g., retinoic acid, retinol)	Wnt/β-catenin and downstream targets	(i) ↑β-catenin degradation in proteasomes via RXR-mediated pathway; (ii) ↓transcription of the TOPFlash reporter and mRNA levels of the cyclin D1 and C-Myc genes	nd	clinical	[178,180]
Specific small molecules	ETC-159	Wnt, PORCN	PORCN inhibitor; effective in treating RSPO-translocation bearing CRC patient-derived xenografts	nd	Phase 1	[196]
	Wnt-974	Wnt, PORCN	Inhibitory effects in metastatic CRC	nd	Phase 1/2	[165]
	LGK-974	Wnt, PORCN	Inhibitory effects in multiple tumor including CRC	nd	Phase 1	[140,165,167]
	Foxy-5	Wnt5 mimicking	Inhibitory effects in multiple tumors including CRC	nd	Phase 1	[165,167]
	G007-LK	Axin	Both are tankyrase inhibitors; both promote β-catenin destabilization; G007-LK inhibits tumor growth in vivo in a subset of APC-mutant CRC xenograft models	nd	preclinical	[169]
	G244-LM					
	LF3 (4-thioureido-benzenesulphonamide derivative)	β-catenin/TCF	(i) antagonises of β-catenin/TCF4 interactions; (ii) suppresses cell motility, cell-cycle progression; (iii) ↓tumor growth and induces differentiation in a mouse xenografts of CRC	nd	discovery	[197]
	SAH-BCL9	Blockade of β-catenin protein-protein interactions	(i) dissociates native β-catenin/BCL9 complexes, selectively suppresses Wnt transcription, and exhibits antitumor effects; (ii) suppresses tumor growth and angiogenesis in mouse xenograft model of CRC	inhibits	preclinical	[83]
	XAV-939	β-catenin, Axin	Inhibits β-catenin which resulted in sensitization of CRC cells to chemotherapy	nd	discovery	[182]
Antibodies	Anti-RSPO3 mAb (Rosmantuzumab, OMP-131R10)	RSPO3 (Wnt agonist)	In PTPRK-RSPO3-fusion positive human colon tumors xenografts inhibits tumor growth and promotes differentiation	nd	Phase 1	[198]
Plant-based agents	Berberine and synthetic 13-arylalkyl derivatives	β-catenin	(i) inhibits β-catenin transcriptional activity by binding to a unique RXRα region; (ii) weakening of anchorage-independent growth (↓E-cadherin expression)	nd	discovery	[192,193,194]
	Bryostatin 1	Wnt/β-catenin	(i) natural PKCα activator; (ii) PKCα triggers the death of CRC cells; (iii) PKCα activity is drug-inducible	nd	Phase 2	[74]
	Curcumin (diferuloymethane)	Wnt/β-catenin and downstream proteins	anti-tumor activity via inhibition of cell proliferation, pro-apoptotic effects, decrease in CDX2 and expression of Wnt3a, c-Myc, survivin, and cyclin D1	nd	Phase 1–3 *	[140,188,189,190]
	Genistein	Wnt/β-catenin and downstream proteins	(i) ↓nuclear β-catenin and increases phospho-β-catenin accumulation; (ii) inhibits cell viability, cell invasion, cell migration by recovering WIF1, ↑apoptosis; (iii) ↑sFRP2 gene expression by demethylating its silenced promoter; (iv) ↓MMP-2 and MMP-9, but ↑E-cadherin	nd	Phase 1–2 *	[199,200]
	Isobavachalcone	AKT/GSK3β/β-catenin pathway	inhibits growth and colony formation of tumor cells, as well as induces apoptosis	nd	discovery	[190]
	Resveratrol (SRT501, grapes)	TCF4	(i) ↓cellular accumulation of endogenously-introduced TCF4 protein; (ii) represses the growth of CRC cells	nd	Phase 1	[140,201]
	RTHF	Wnt/β-catenin	↓β-catenin and downstream protein expression (Lgr5, c-Myc, and cyclin D1)	nd	discovery	[187]
	Thymol	Wnt/β-catenin	(i) prevents EMT, invasion, and CRC metastasis	nd	discovery	[186]

Abbreviations: ↑,↓—increase (up-regulation)/decrease expression/level; *—used also in combination with radiation therapy and chemotherapy; ANG—Angiopoietin; bFGF—(basic) Fibroblast Growth Factor; CDX2—Caudal Type Homeobox-2; COX-2—Cyclooxygenase-2; EMT—Epithelial-Mesenchymal Transition; GSK3β—Glycogen Synthase Kinase 3β; HUVECs—Human Umbilical Vein Endothelial Cells; nd—not determined; MMPs—Matrix Metalloproteinases; NSAIDs—non-steroidal anti-inflammatory drugs; PKCα—Protein Kinase C α; PORCN—Porcupine; PTPRK—Receptor-type Tyrosine-protein Phosphatase kappa; RSPO1-4—Wnt agonists of the R-spondin family; RTHF—Radix *Tetrastigma hemsleyani* flavone; RXR—retinoid X receptor; sFRP2—secreted Frizzled related protein 2; TCF4—Transcription Factor 4, T-cell Factor-4; TOPFlash—TCF Reporter Plasmid; WIF1—Wnt Inhibitory Factor 1.

## Abbreviations

aa	Amino acids
Akt/AKT	Serine-threonine Protein Kinase (PKB, now called AKT1)
ANG-1, -2	Angiopoietin-1, -2
APC	Adenomatous Polyposis Coli
(b)FGF	(basic) Fibroblast Growth Factor
Bcl-2	B-cell lymphoma protein
BCL9	B cell CLL/lymphoma 9 protein
CDX	Caudal Type Homeobox-2 Protein
CD44, 184	Cluster of Differentiation 44, 184
COX-2	Cyclooxygenase-2
CRC	Colorectal Cancer
CXCL8	C-X-C motif ligand 8 (chemokine)
CXCR4	C-X-C chemokine receptor type 4
DKK-1, -4	Dickkopf-related protein 1;-4
ECs	Endothelial Cells
ECM	Extracellular Matrix
EMT	Epithelial-Mesenchymal Transition
ERK1/2	Extracellular Signal-regulated Kinase ½
FAK	Focal Adhesion Kinase
FOXQ1	Forkhead Box Q1 Protein
FRAT1	Frequently Rearranged in Advanced T-cell Lymphoma 1 Protein
FRPs	Fzd-related Proteins
Fzd	Frizzleds proteins, a family of G protein-coupled receptor proteins
GDF-15	Growth Differentiation Factor 15
GSK3β	Glycogen Synthase Kinase 3 β
HIF-1α	Hypoxia-inducible Factor 1 α
HUVECs	Human Umbilical Vein ECs
IL	Interleukin
KRAS	Kirsten Rat Sarcoma Virus, proto-oncogene
LEF	Lymphoid Enhancer Factor
MAPK	A Mitogen-activated Protein Kinase
MMP-2, -9	Matrix Metalloproteinase 2, 9
MVD	Microvessel Density
PI3K	Phosphatidylinositol 3’ Kinase
PKA, B (AKT), C α	Protein Kinase A, B (AKT), C α
PKG	Type 1 cyclic Guanosine Monophosphate (cGMP)-dependent Protein Kinase
PLCγ1	Phospholipase C γ1
p-LPR6	Phosphorylated Lipoprotein-related Protein 6
ROS	Reactive Oxygen Species
RAR	Retinoic Acid Receptor
RORα	RAR-related Orphan Receptor α;
RYK	Related to Receptor Tyrosine Kinase protein
SALL4	Zink Finger Transcription Factor Spalt (Sall)-like Protein 4
SFRP2	Secreted Frizzled-related Protein 2
SMAD4	SMAD family member 4, Mothers Against Decapentaplegic Homolog 4
SMAR1	Scaffold/Matrix Attachment Region Binding Protein 1
STAT3	Signal Transducer and Activator of Transcription Protein Activator of Transcription 3
Tan IIA, TSA	Tanshinone IIA
TCF	T cell Factor, Transcription Factor
TCF7L2	Transcription Factor 7-like 2
TGF-β	Tumor Growth Factor beta
TGM2	Tissue Transglutaminase 2
TIPE2 (TNFAIP8L2)	TNFα-induced protein 8 like 2
TNF-α	Tumor Necrosis Factor α
TNM	T—tumor; N—lymph nodes; M—metastasis
TOPFlash	TCF Reporter Plasmid
TP53	Tumor Protein 53
3’UTR	3’ Untranslated Region
VEGF	Vascular Endothelial Growth Factor
VEGF (R)	Vascular Endothelial Growth Factor (Receptor)
